# Understanding the Chemosensory and Detoxification Mechanisms in the Oriental Fruit Fly, *Bactrocera dorsalis*

**DOI:** 10.3390/insects17040416

**Published:** 2026-04-14

**Authors:** Saleem Jaffar, Yongyue Lu

**Affiliations:** Department of Entomology, College of Plant Protection, South China Agricultural University, Guangzhou 510642, China

**Keywords:** Diptera, *Bactrocera dorsalis*, methyl eugenol, transcriptome, chemosensory genes, detoxification genes

## Abstract

*Bactrocera dorsalis* (Hendel) is a major fruit-feeding pest that poses a severe and persistent threat to the horticulture industry in tropical and subtropical regions. Methyl eugenol (ME) is a powerful male-specific attractant phytochemical and pheromone precursor that has been widely exploited in lure-and-kill pest management programs. Upon ingestion, ME is metabolized (*E*)-coniferyl alcohol (E-CF) and 2-allyl-4,5-dimethoxyphenol (DMP), which are stored in the male rectal glands and released during courtship to attract females. Despite its ecological significance, the fundamental molecular mechanism underlying ME perception remains poorly understood. Here, we performed a comparative transcriptomic analysis of ME-responsive and ME-non-responsive male *B. dorsalis* across four tissues (head, gut, midleg, and wing). A total of 15,727 genes were annotated, of which 970 were associated with odorant-binding proteins (OBPs), odorant receptors (ORs), gustatory receptors (GRs), ionotropic receptors (IRs), and chemosensory proteins (CSPs), as well as detoxification families comprising cytochrome P450s (CYPs), carboxylesterases (CaEs), glutathione *S*-transferases (GSTs), and uridine diphosphate (UDP)-glycosyltransferases (UGTs), and the stress-related heat shock proteins (HSPs) genes. Differential expression analysis identified 7222, 7763, and 6105 differentially expressed genes (DEGs) in the head, gut, and wings/midlegs, respectively, between ME-responsive and ME-non-responsive males. Notably, CYPs, UGTs, and HSPs involved in detoxification and stress response were significantly downregulated. Gene Ontology (GO) and Kyoto Encyclopedia of Genes and Genomes (KEGG) enrichment analyses revealed that CYPs were significantly enriched in metabolic detoxification pathways. These findings reveal a complex molecular interplay between olfaction and detoxification and suggest that ME induces coordinated genetic pathways supporting survival, reproduction, and environmental adaptability. This knowledge provides a foundation for the development of eco-friendly pest management strategies targeting these molecular mechanisms.

## Simple Summary

The oriental fruit fly, *Bactrocera dorsalis*, is a globally important agricultural pest. Male flies are strongly attracted to the plant-derived compound methyl eugenol (ME), which they ingest and metabolize into sex pheromone precursors. In this study, transcriptome sequencing was used to investigate how flies detect and metabolize ME following exposure. Thousands of genes associated with chemosensory perception and detoxification were identified across multiple tissues. These findings provide new insights into the molecular mechanisms underlying ME attraction and metabolism and may contribute to the development of targeted and environmentally friendly pest management strategies.

## 1. Introduction

Plasticity in sensory perception and xenobiotic metabolism is fundamental to the ecological adaptation and evolutionary success of insects [1,2]. In insects, the chemosensory system detects environmental chemical cues, while detoxification pathways metabolize and eliminate harmful compounds. Together, these systems enable adaptation to diverse ecological niches and host-associated chemicals. The chemosensory system comprises odorant receptors (ORs), ionotropic receptors (IRs), gustatory receptors (GRs), and odorant-binding proteins (OBPs), which collectively enable insects to detect and respond to a diverse chemical environment [3,4]. In parallel, the detoxification system, mediated by enzyme superfamilies such as cytochrome P450s (CYPs), glutathione S-transferases (GSTs), carboxylesterases (CAEs), and UDP-glucuronosyltransferases (UGTs), protects insects from a range of xenobiotics. [5,6]. Stress-responsive heat shock proteins (HSPs) also play an important role in maintaining cellular homeostasis under physiological and chemical stress. Importantly, these systems are functionally interconnected: chemosensory perception often guides exposure to chemical stimuli, which in turn triggers metabolic and stress responses to maintain homeostasis [7,8,9,10,11]. Such integrated responses are increasingly important in the context of insecticide resistance, as insects evolve both behavioral and biochemical strategies to cope with toxic compounds.

The oriental fruit fly, *Bactrocera dorsalis* (Diptera: Tephritidae) (Figure S1A,B), is a highly invasive and polyphagous pest attacking over 250 species of fruits and vegetables [12,13]. Its global spread and substantial economic impact have made it a major target for area-wide pest management programs, which traditionally rely on chemical insecticides [14,15]. The extensive use of organophosphates, pyrethroids, and spinosyns has driven the emergence of significant resistance in *B. dorsalis* populations [16,17]. In parallel, males display a unique behavioral response to the plant-derived phenylpropanoid methyl eugenol (ME), which is exploited in “lure-and-kill” strategies worldwide: males attracted to ME ingest it, and the compound is metabolized and converted into sex pheromone components, enhancing their mating success (Figure S2) [18,19,20,21]. ME ingestion represents a xenobiotic challenge that demands a robust detoxification response, and the molecular mechanisms underlying this response involve coordinated activity of both chemosensory and detoxification systems.

Specific ORs and OBPs are tuned to ME detection, guiding male feeding behavior [22]. Once ingested, oxidative and conjugative enzymes, including CYPs, GSTs, and ESTs, metabolize ME into downstream compounds such as DMP and ECF [10,23]. This metabolic processing may induce cellular stress, triggering HSP-mediated stress to maintain protein homeostasis [24,25]. Interestingly, exposure to xenobiotics can also modulate chemosensory gene expression, potentially altering olfactory-guided behaviors [26,27]. These observations suggest an integrated sensory and metabolic system in which chemical detection directly influences detoxification capacity, and vice versa.

Male-specific ORs, including the conserved co-receptor Orco and its tuning partners, are essential for ME detection and intraspecific communication, while IRs and GRs mediate sensing of acids, amines, and sugars, guiding feeding and oviposition decisions in females [28,29,30,31,32,33]. Beyond their canonical roles, emerging evidence indicates that chemosensory proteins and detoxification enzymes can exhibit overlapping or dual functions. For example, CYPs traditionally associated with xenobiotic metabolism may also facilitate sequestration or transport of plant-derived compounds within the body [34,35].

Despite prior studies, a comprehensive, manually curated catalogue of chemosensory and detoxification genes for *B. dorsalis* has been lacking [36,37], limiting functional understanding in this economically important Tephritidae species. Key questions persist regarding which specific chemosensory genes are responsible for the detection of ME and how detoxification mechanisms are coordinated with chemosensory responses. Although several key genes, including *BdorOBP2*, *BdorGOBP69a*, *BdorOR49b*, *BdorOR59a*, *BdorOR69a*, *BdorOBP56f*-2, *BdorOR83a*, and *BdorOR88a*, have been identified as relevant to ME responses, their interplay with the detoxification pathway remains unclear. Such manual curation has been performed in other insect pests, including American cockroach (*Periplaneta americana*) [38] and Drosophilidae [39], but a comprehensive, manually verified repertoire is lacking for *B. dorsalis*.

Among the best-characterized resistance mechanisms in insect pests are target-site mutations, enhanced metabolic detoxification, and cuticular modifications limiting insecticide penetration [40,41]. Increasing evidence implicates CSPs and OBPs as key mediators of insecticide sequestration, contributing to resistance phenotypes [38,42]. Frequently, resistant mechanisms exhibit altered CSP and OBP expression [43], which is further induced upon insecticide exposure [44,45,46]. Functional validation through knockdown approaches has confirmed that members of both protein families participate in sequestration and confer protection against insecticides across multiple insect species [42,47,48,49,50,51,52].

CSPs and OBPs are small (~10–20 kDa), soluble, globular proteins stabilized by conserved disulfide bonds [3,53]. Their compact fold typically comprises four CSPs or six (classic OBPs) α-helices that enclose a central hydrophobic binding pocket. OBPs are classified into subfamilies according to cysteine signature and domain architecture: Classic OBPs possess six conserved cysteines forming three interlocking disulfide bonds; Plus-C OBPs contain two additional cysteines and a conserved proline; Minus-C OBPs lack two of the six signature cysteines; Atypical OBPs have 9–10 cysteines and a prolonged C-terminal region; and Dimer OBPs consist of two classic OBP domains [39,54,55,56].

To address these challenges, we investigated the genome-wide transcriptome response of ME in male *B. dorsalis* head, gut, midleg, and wing. We manually curated key gene families, including ORs, IRs, GRs, OBPs, and CSPs (chemosensory), as well as HSPs, CYPs, GSTs, CAEs, and UGTs (detoxification) from the available *B. dorsalis* genome. Using RNA-seq, we quantified their expression changes between ME-treated and control males across tissues. These gene families were subjected to comprehensive annotation, molecular phylogenetic analysis, and expression profiling. This study provides a systems-level understanding of the molecular events triggered by ME exposure, offering insights that could inform the development of next-generation behavioral attractants and resistance management strategies.

## 2. Materials and Methods

### 2.1. Sample Origin and Preparation

The oriental fruit fly *B. dorsalis* population was reared in the laboratory at the College of Plant Protection, South China Agricultural University. Adults and larvae were maintained on an artificial diet following Fan et al. [57]. A highly inbred line of *B. dorsalis* (>30 generations of sibling mating) was used to minimize genetic variation and ensure experimental reproducibility. For transcriptomic analyses, 300 unmated adult males were selected, providing a genetically uniform population. Flies were reared under controlled conditions at 27 °C ± 2 °C and 60–70% relative humidity. Mature males (10–16 days old) were starved for 12 h prior to treatment, following the methodology of Liu et al. [58].

Flies were divided into two experimental groups: a treatment group exposed to ME (Sigma-Aldrich, St. Louis. USA, purity > 98%), and a control group exposed to mineral oil (Sigma-Aldrich) as a neutral carrier solvent. Exposure was performed under controlled laboratory conditions (Figure S3). At 96 h post-exposure to 1 mL of ME [57], tissues, including heads, guts, midlegs, and wings, were collected separately from flies exhibiting continued feeding on ME (ME-responsive) and those that had ceased feeding (ME-non-responsive).

**Figure 1:**
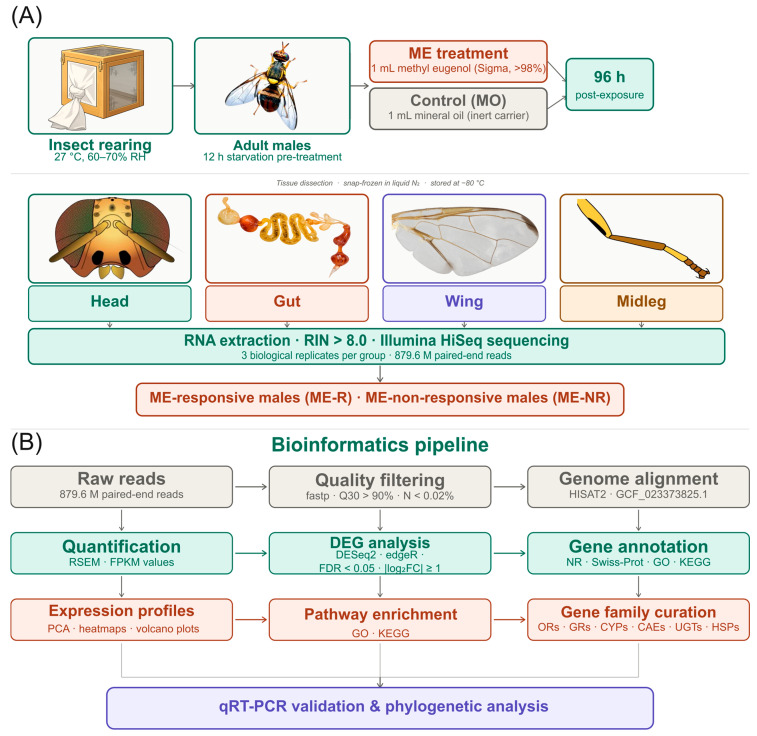
Experimental design and bioinformatics pipeline for transcriptomic analysis of male *Bactrocera dorsalis* in response to methyl eugenol (ME) exposure. (**A**) Adult males were exposed to ME or mineral oil control (MO) for 96 h and classified as ME-responsive (ME-R) or ME-non-responsive (ME-NR). Head, gut, wing, and midleg tissues were dissected and sequenced on the Illumina HiSeq platform. (**B**) Reads were aligned to the *B. dorsalis* reference genome (GCF_023373825.1), quantified by RSEM, and DEGs were identified using DESeq2 and edgeR (FDR < 0.05; |log_2_FC| ≥ 1). Functional annotation, pathway enrichment, gene family curation, and qRT-PCR validation were performed as described in the Methods.

All dissected tissues were immediately snap-frozen in liquid nitrogen and stored at −80 °C until RNA extraction, following Liu et al. [58]. Total RNA was isolated from pooled tissue samples using standard protocols, and RNA integrity was assessed using an Agilent Bioanalyzer, with all samples exhibiting RIN values >8.0, confirming high-quality RNA suitable for downstream library construction. This approach ensured both tissue-specific and behavioral-state-specific transcriptomic profiling, enabling robust comparisons. The overall experimental design and downstream bioinformatics workflow are summarized in Figure 1.

### 2.2. Transcriptome Sequencing and Assembly

High-quality RNA libraries were prepared from the four tissue types. Short-read libraries were sequenced on the Illumina NovaSeq 6000 platform (Gene Denovo Biotechnology, Guangzhou, China). Raw Illumina reads were quality-filtered using fastp v0.18.0 [59], to remove (i) adapters, (ii) reads with >10% ambiguous bases (N), and (iii) low-quality reads with >50% of bases having Q ≤ 20. Transcriptomic reads were aligned to the draft *B. dorsalis* reference genome using HISAT2 v2.4 [60], and transcript structures were reconstructed using StringTie v1.3.1 [61,62]. De novo transcript assembly was performed using Trinity v2013-02-25 [63], followed by transcript clustering with TGICL v2.1 [64]. Integration of reference-based and de novo assemblies provided transcriptional support for an accurate gene prediction model.

### 2.3. Functional Annotation

**Figure 2:**
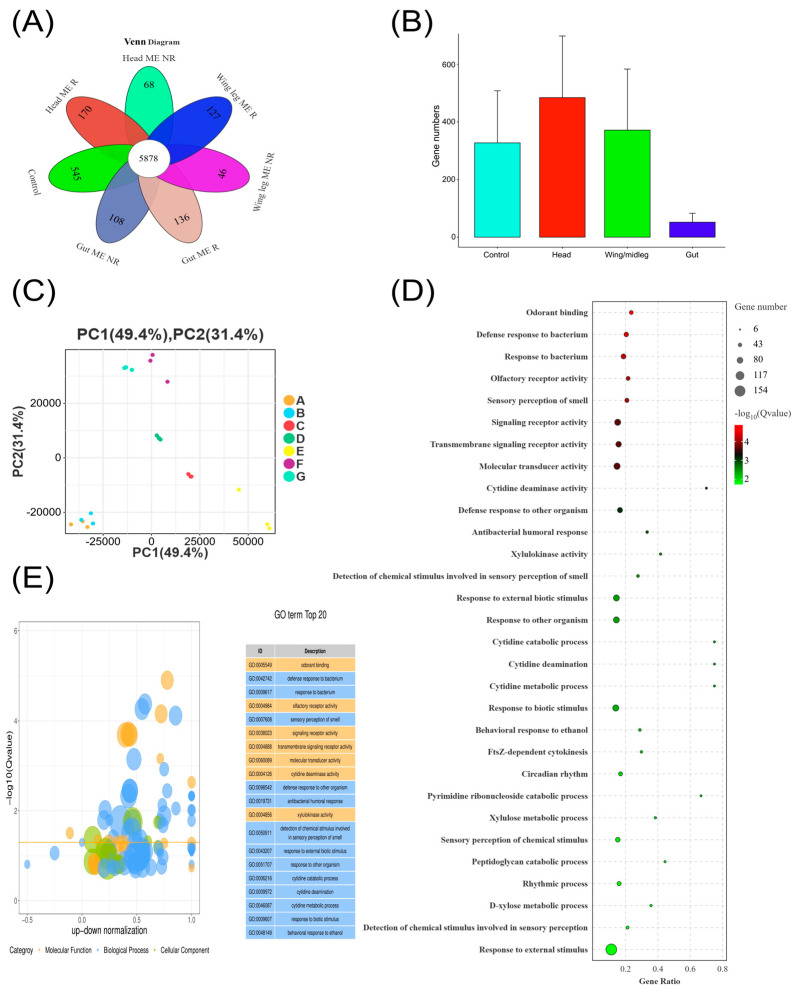
Overview of differentially expressed genes (DEGs) in male *Bactrocera dorsalis* following methyl eugenol (ME) exposure. (**A**) Venn diagram showing the number of genes expressed exclusively or commonly across seven sample groups: Head ME-responsive (ME-R), Head ME-non-responsive (ME-NR), Wing/Leg ME-R, Wing/Leg ME-NR, Gut ME-R, Gut ME-NR, and MO control. The central value (5878) represents genes shared across all groups. (**B**) Mean number of DEGs per tissue group (control, head, wing/midleg, gut). (**C**) Principal component analysis (PCA) of normalized gene expression profiles. Each point represents one biological replicate; PC1 and PC2 explain 49.4% and 31.4% of total variance, respectively. Sample groups: A = Head ME-R, B = Head ME-NR, C = Gut ME-R, D = Gut ME-NR, E = Wing/Leg ME-R, F = Wing/Leg ME-NR, G = MO control. (**D**) Gene Ontology (GO) enrichment dot plot showing the top 30 enriched terms. Dot size represents gene count; color represents −log_10_ (Q-value). (**E**) GO enrichment bubble plot (left) and top 20 GO term table (right). Bubble position represents up–down proportional enrichment; color indicates GO category (orange: molecular function; blue: biological process; green: cellular component). Horizontal line indicates Q-value = 0.05 threshold. All enrichment analyses applied Bonferroni correction (*p* ≤ 0.05).

Gene prediction combined (a) ab initio models, (b) RNA-seq-supported structures, and (c) protein homology from Dipteran species. Functional annotation of predicted unigenes was performed using BLASTX. 2.16.0 (E-value < 1 × 10^−5^) against NCBI non-redundant (Nr), Swiss-Prot, Kyoto Encyclopedia of Genes and Genomes (KEGG), and Gene Ontology (GO) databases. GO terms were assigned using Blast2GO. 6.0 (Blast2GO—Functional Annotation and Genomics), categorizing genes into biological process, molecular function, and cellular component (Figure 2).

### 2.4. Comparative Analyses

**Figure 3:**
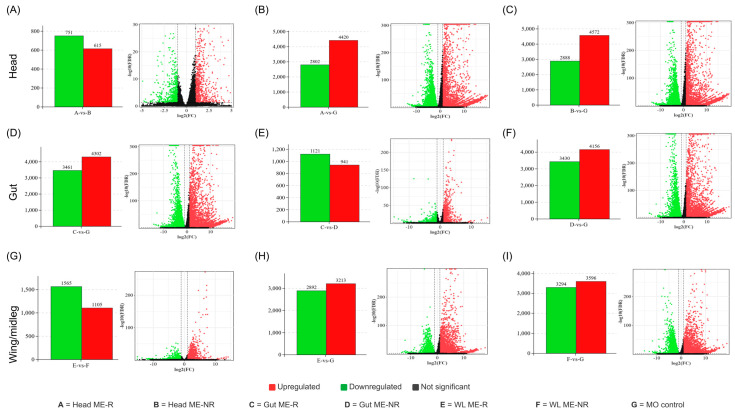
Volcano plots and DEG counts for all nine pairwise comparisons across head, gut, and wing/leg tissues in male *Bactrocera dorsalis* following methyl eugenol (ME) exposure. Each panel pair comprises a bar chart (left) showing the number of downregulated (green) and upregulated (red) DEGs, and a volcano plot (right). A = Head ME-responsive (ME-R); B = Head ME-non-responsive (ME-NR); C = Gut ME-R; D = Gut ME-NR; E = Wing/Leg ME-R; F = Wing/Leg ME-NR; G = mineral oil control (MO). Figure panels: (**A**) Head ME-R vs. Head ME-NR; (**B**) Head ME-R vs. MO control; (**C**) Head ME-NR vs. MO control; (**D**) Gut ME-R vs. MO control; (**E**) Gut ME-R vs. Gut ME-NR; (**F**) Gut ME-NR vs. MO control; (**G**) Wing/Leg ME-R vs. Wing/Leg ME-NR; (**H**) Wing/Leg ME-R vs. MO control; (**I**) Wing/Leg ME-NR vs. MO control. In all volcano plots: red dots = upregulated DEGs; green dots = downregulated DEGs; black dots = not significant.

Comparative genomic analyses used the *B. dorsalis* gene set (v0.6.2) alongside orthologous sequences from *Drosophila melanogaster* and other Diptera. Gene families of interest, including chemosensory receptors (ORs, GRs, IRs), detoxification enzymes (CYPs, GSTs, ESTs, UGTs), and stress-response genes (HSPs), were systematically re-annotated using a uniform pipeline to minimize interspecies bias. Homolog identification employed BLASTP with a minimum amino acid identity of 30%. Comparative counts were visualized to highlight expansions or contractions in *B. dorsalis* (Figure 3).

### 2.5. Gene Family Curation: Chemosensory and Detoxification

Approximately 2000 candidate genes linked to chemosensory detection and xenobiotic metabolism were manually curated. Gene family definitions were established using *Drosophila* homologs. Candidate OBPs and CSPs were retrieved from GenBank (https://www.ncbi.nlm.nih.gov/genbank/ (accessed on 1 March 2025)), FlyBase (https://flybase.org/ (accessed on 15 March 2025)), and the i5k *B. dorsalis* protein dataset with sequence homology verified using BLASTP *NCBI*. Antennal transcriptome data (GenBank SRR9026238) were used for final validation, orthology, and phylogenomics.

High-confidence orthologs across Dipteran species were identified via BLASTP and curated gene sets. Protein sequences were aligned using ClustalX v2.2.0, poorly aligned regions were removed, and maximum-likelihood phylogenies were inferred with MEGA11 https://itol.embl.de/ (accessed on 25 March 2025) under the JTT model with 100 bootstrap replicates. These analyses identified lineage-specific expansions and evolutionary trends in chemosensory and detoxification gene families [65].

### 2.6. Gene Expression Validation

Sixteen olfactory receptors were selected for qRT-PCR validation. Primers were designed using Primer3 (https://primer3.ut.ee/ (accessed on 1 April 2025 )) (Table S1). qRT-PCR reactions were prepared using SYBR Premix Ex Taq (Tiangen, China) on a Stratagene Mx3000P system (Agilent Technologies Guangzhou, China). Amplification specificity was confirmed by dissociation curve analysis. α-tubulin (GenBank GU269902) served as the reference gene. Normalized expression levels were calculated using the 2^−ΔΔCT^ method [66].

### 2.7. Statistical Analyses

All statistical analyses were conducted in SAS v9.20. Data are presented as mean ± SEM. Student’s *t*-tests were used for comparisons between treatment groups, with significance set at *p* < 0.05.

## 3. Results

### 3.1. Overview of RNA Sequencing

Illumina HiSeq seq sequencing of six libraries generated a total of 39.54 GB of high-quality clean data, yielding 879,609,600 aligned reads. All libraries met stringent quality thresholds: Q20 and Q30 values exceeded 97.0% and 90.0%, respectively, and the average Q30 percentage across samples was 93.82%, confirming high basal accuracy. The mean GC content was 44.83%, and the proportion of ambiguous bases (N) was below 0.02% across all clean reads, indicating minimal sequencing error. GC content variation across individual libraries (ranging from 36.33% to 40.87%) is consistent with tissue-specific differences in transcript composition across head, gut, wing, and midleg samples rather than treatment effects per se, as comparisons of GC content are only interpretable within the same tissue type.

Clean reads were aligned to the *B. dorsalis* reference genome (GCF_023373825.1) using HISAT2. Mapping rates ranged from 12.31% to 89.48% across libraries (Tables S2 and S3). The lower mapping rates observed in wing/leg libraries are consistent with incomplete representation of non-head, non-gut tissues in the current reference annotation and do not reflect reduced sequencing quality. A total of 12,602 unigenes (96.3% of annotated transcripts) were successfully matched to at least one public database entry, confirming broad functional coverage of the transcriptome. All datasets have been deposited in the GenBank Sequence Read Archive (BioProjects No. PRJNA1102686, PRJNA1102347, PRJNA1102690, and PRJNA1102693) (Table S4).

KEGG analysis showed that a total of 36,599 unigene sequences were classified into 35 functional categories. The largest category was the odorant binding group (36; 4.91%), followed by the defense response mechanisms group (47; 4.38%), and olfactory receptor activity (36; 4.16%).

### 3.2. Differentially Expressed Genes and Functional Annotation

To identify DEGs, we employed FPKM-based normalized expression values across different tissues. The overlap of expressed genes across all seven sample groups—Head ME-R, Head ME-NR, Wing/Leg ME-R, Wing/Leg ME-NR, Gut ME-R, Gut ME-NR, and MO control—is shown in Figure 2A. A total of 5878 transcripts were shared across all groups, representing the constitutively expressed core transcriptome of male *B. dorsalis*, while tissue- and response-state-specific transcripts formed distinct non-overlapping sets.

A total of 7222 DEGs were identified in the head, 7763 in the gut, and 6105 in the wing/legs, using criteria of |log_2_FC| > 1 and both *p*-value and FDR < 0.05. Specifically, in the head, 2802 DEGs (38.79%) were downregulated, while 4420 (61.20%) were upregulated. In the gut, 3461 DEGs (44.58%) were downregulated and 4302 (55.41%) upregulated. In the WL, 2892 DEGs (47.37%) were downregulated, and 3213 DEGs (52.62%) were upregulated in treated males. These results indicate profound transcriptomic reprogramming induced by ME exposure and provide valuable insight into the gene regulatory mechanisms. Principal component analysis confirmed clear, reproducible separation of ME-treated and MO control groups across all tissues, with biological replicates clustering tightly within each treatment group (Figure 2C). Gene Ontology enrichment analysis of DEGs across all tissues identified significant enrichment of terms related to odorant binding, olfactory receptor activity, sensory perception of smell, and defense response (Figure 2D). The predominance of chemosensory and immune-related GO terms among the most significantly enriched categories reflects the dual nature of ME as both a semiochemical attractant and a xenobiotic compound requiring detoxification. GO bubble plot analysis further confirmed enrichment of oxidoreductase and receptor signaling activity terms, with the top 20 enriched GO terms including odorant binding, signaling receptor activity, and molecular transducer activity (Figure 2E). KEGG pathway enrichment analysis identified significant enrichment of metabolic pathways, drug metabolism enzymes, MAPK signaling, and Toll and Imd innate immune signaling among the top enriched pathways across tissues (Figure S4).

The global distribution of DEG significance and fold-change magnitude across all nine pairwise comparisons is visualized as volcano plots in Figure 3. Comparisons between ME-treated groups and the MO control consistently yielded the largest DEG sets: Head ME-R vs. MO control (2802 downregulated, 4420 upregulated; Figure 3B), Gut ME-R vs. MO control (3461 downregulated, 4302 upregulated; Figure 3D), and Wing/Leg ME-R vs. MO control (2892 downregulated, 3213 upregulated; Figure 3H). ME-NR males similarly showed extensive transcriptional divergence from the MO control: Head ME-NR vs. MO control (2888 downregulated, 4572 upregulated; Figure 3C), Gut ME-NR vs. MO control (3430 downregulated, 4156 upregulated; Figure 3F), and Wing/Leg ME-NR vs. MO control (3294 downregulated, 3596 upregulated; Figure 3I), confirming that even non-feeding males undergo substantial transcriptional reprogramming relative to the unexposed baseline.

Within-tissue comparisons between ME-R and ME-NR males revealed smaller but significant DEG sets, reflecting transcriptional differences attributable specifically to continued feeding behavior. The head ME-R vs. ME-NR comparison (Figure 3A) yielded 751 downregulated and 615 upregulated DEGs, with a notably compressed fold-change range (|log_2_FC| < 5) and lower significance scores compared to treatment-vs-control comparisons, indicating that head transcriptomes of ME-R and ME-NR males are more similar to each other than either is to the unexposed control. The gut ME-R vs. ME-NR comparison (Figure 3E) produced 1121 downregulated and 941 upregulated DEGs, the largest within-tissue ME-R vs. ME-NR set, with fold changes extending to |log_2_FC| ~10, consistent with the gut being the organ most directly influenced by differences in ME consumption between the two groups. The Wing/Leg ME-R vs. ME-NR comparison (Figure 3G) yielded 1565 downregulated and 1105 upregulated DEGs, suggesting that peripheral tissue transcriptomes also reflect feeding-state differences, possibly through systemic hormonal or neurochemical signals downstream of ME ingestion.

### 3.3. Chemosensory Gene Expression Profiles

Chemosensory receptor genes were identified through BLAST searches against the *B. dorsalis* genome, guided by *Drosophila* homologs, with hypothetical proteins and low-confidence predictions excluded. In total, 86 ORs were identified across the genome annotation (Tables S5 and S6). Significant expression changes were observed in the heads of ME-treated males compared to controls, with 15 ORs upregulated and 71 downregulated. Among the upregulated ORs, *BdorOr7a*, *BdorOr22c*, and *BdorOr85d* showed fold changes of 6.5, 5.8, and 4.5, respectively. Conversely, *BdorOr63a*, *BdorOr69a*, *BdorOr30a*, *BdorOr94a*, and *BdorOr46a* were among the most downregulated, with fold changes of −7.5, −7.5, −7.6, −7.3, and −7.9, respectively. The predominance of OR downregulation in ME-R males suggests broad olfactory adaptation following sustained ME exposure, consistent with a negative feedback mechanism attenuating receptor-level sensitivity after initial detection.

**Figure 4:**
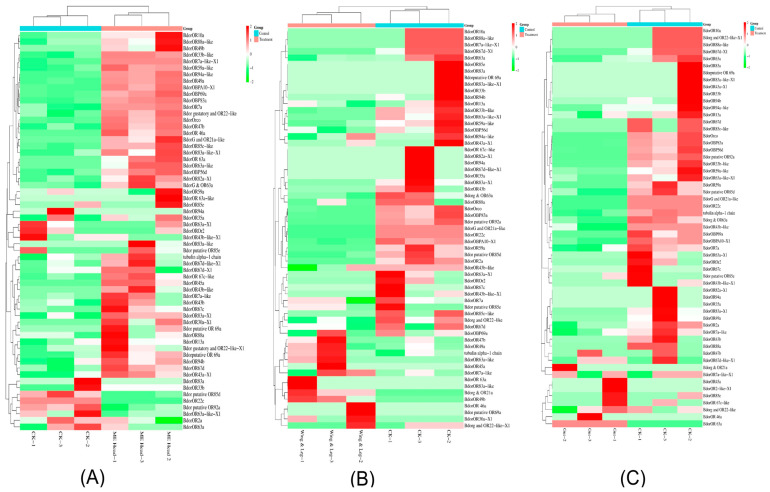
Heatmaps showing differential expression patterns of chemosensory-related genes across (**A**) head, (**B**) wing/leg, and (**C**) gut tissues. Color gradients indicate relative expression levels following normalization, where red denotes high expression, green denotes low expression, and intermediate shades indicate moderate expression levels.

In ME-NR males, a distinct OR regulation pattern was observed. *BdorOR59-like-3* (9.2-fold) and *BdorOR21a-like* (7.6-fold) were among the most upregulated ORs, suggesting partial restoration of olfactory receptor transcription in non-feeding males. In contrast, *BdorOR63a* (−8.03-fold), *BdorOR45a-1* (−7.5-fold), and *BdorOR69a* (−6.9-fold) remained markedly downregulated, an overlapping set with the receptors suppressed in ME-R males. This indicates that suppression of these specific ORs persists regardless of continued feeding behavior. The expression profile of ORs across different tissues is shown in Figure 4. Together, the ME-R and ME-NR OR profiles reveal a complex, state-dependent reconfiguration of the olfactory receptor repertoire following ME exposure.

**Figure 5:**
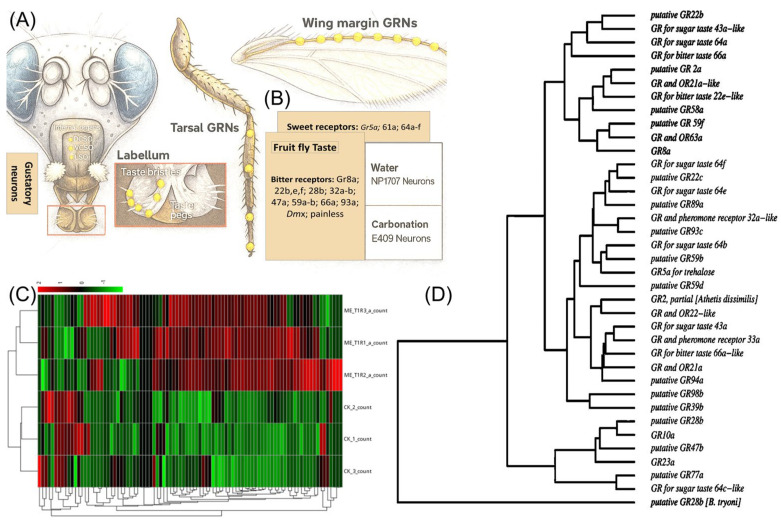
Chemosensory structures, receptor classification, and expression overview in *Bactrocera dorsalis.* (**A**) Schematic illustration of gustatory receptor neurons (GRNs) in *B. dorsalis*, highlighting major chemosensory organs including the labellum, tarsal segments, and wing margin. (**B**) Classification of GR families identified in *B. dorsalis*. (**C**) Heatmap showing gene expression profiles of GRs. (**D**) Phylogenetic tree of GR proteins from *B. dorsalis* and related species, illustrating evolutionary relationships and classification into subfamilies.

In addition to ORs, 63 GRs were identified, showing tissue-specific differential expression (Table S7). In the head, *BdorGR22e1*, *BdorGR22e2*, and *BdorGR22e3* were strongly induced (8.2-, 4.9-, and 6.9-fold, respectively), while *BdorGR22c*, *BdorGR39b-3*, and *BdorGR36c* were the most downregulated (−6.4-, −6.3-, and −5.0-fold, respectively). The upregulation of *GR22e-like* transcripts, encoding bitter taste receptors, may reflect an aversive gustatory signal associated with high ME concentrations or its metabolites, as bitter taste receptors commonly respond to potentially toxic compounds. Conversely, downregulation of sugar and pheromone-associated GRs (LOC105224525, *GR43a*, −4.57-fold; LOC105229347, *GR32a*, −2.41-fold) may indicate suppression of competing food-search and mate-search behaviors following ME satiation. A substantial subset of GR genes (15 transcripts) showed no expression change (log_2_FC = 0, FDR = 1), consistent with constitutive expression in tissue types not sampled at the relevant developmental stage. In wing/leg tissue, GR expression also showed significant regulation, consistent with the presence of tarsal chemoreceptors mediating contact chemosensation. The wing/leg GR expression profile is shown in Figure 5.

### 3.4. Detoxification Gene Expression Profiles

**Figure 6:**
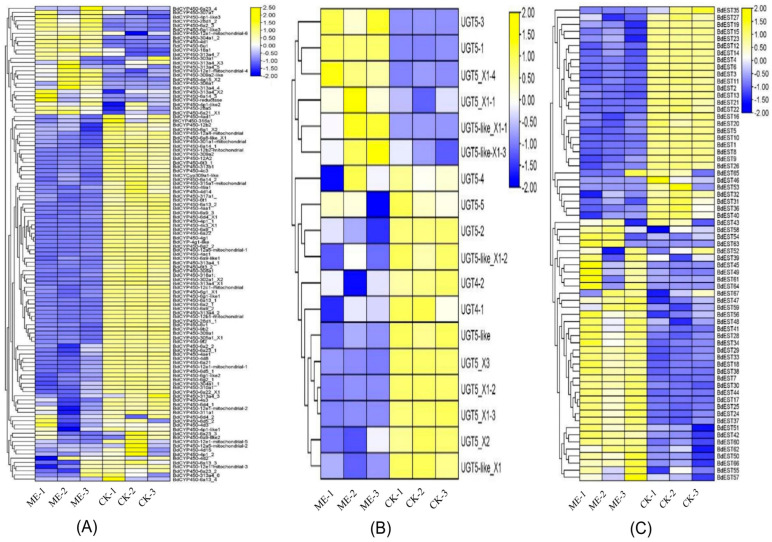
Differential expression of detoxification-related genes in response to methyl eugenol (ME) treatment. (**A**) Heatmap showing expression profiles of CYP (cytochrome P450), (**B**) UGT (UDP-glucuronosyltransferase) genes, and (**C**) EST (esterase) genes illustrating differential expression patterns associated with ME exposure. In all panels, rows represent genes and columns represent biological replicates. Values are normalized expression levels.

Significant changes in the expression of three major detoxification enzyme families, including CYPs, CaEs, and UGTs, were observed in the gut transcriptome of both ME-R and ME-NR male *B. dorsalis* (Figure 6; Tables S8–S10). A total of 114 CYP genes were detected in the ME-R gut transcriptome, with 88 upregulated and 26 downregulated relative to MO controls. The most strongly induced CYPs were *CYP309a2*, *CYP313a4*, *CYP6a22*, *CYP4aa1*, and *CYP4d14*, with fold inductions of 11.4, 11.4, 11.1, 10.8, and 9.2, respectively. The predominance of CYP6 and CYP4 family members among the most highly induced genes is consistent with their established roles in Phase I oxidative metabolism of phenylpropanoids and insecticides across Diptera. Eighteen UGT genes showed differential expression in the ME-R gut; seventeen were upregulated, and one was downregulated. The most highly induced UGTs were UGT5-3 (7.2-fold) and UGT5-like (5.0-fold), indicating activation of Phase II glucuronidation conjugation. Additionally, 67 CaE genes were differentially expressed, with 33 upregulated and 34 downregulated. Juvenile hormone esterase (*BdEst16*, 14.8-fold) and *esterase B1* (*BdEst25*, 8.8-fold) were the most strongly upregulated CaEs, whereas *carboxylesterase 4* (*BdEst59*) was downregulated by 8.2-fold.

In ME-NR gut tissue, 111 CYP genes were differentially expressed, with 68 upregulated and 43 downregulated. The most significantly upregulated CYPs were *BdCYP450-309a1* (12.6-fold), *BdCYP450-6t* (9.3-fold), and *BdCYP450-12a4* (9.1-fold), a partially overlapping but distinct set from those induced in ME-R males, suggesting that CYP induction is not strictly dependent on active feeding behavior. Eighteen UGT genes were differentially expressed in ME-NR gut (twelve upregulated, six downregulated); *UGT5_X2* (7.5-fold) and *UGT5_X3* (6.6-fold) were the most highly upregulated. Among 68 CaE genes showing transcriptional regulation, *BdEst35* (carboxylesterase 5a, 9.3-fold) and *BdEst22* (juvenile hormone esterase, 8.7-fold) were the most upregulated CaEs (Figure 6).

### 3.5. Stress-Response Gene Expression Profiles

**Figure 7:**
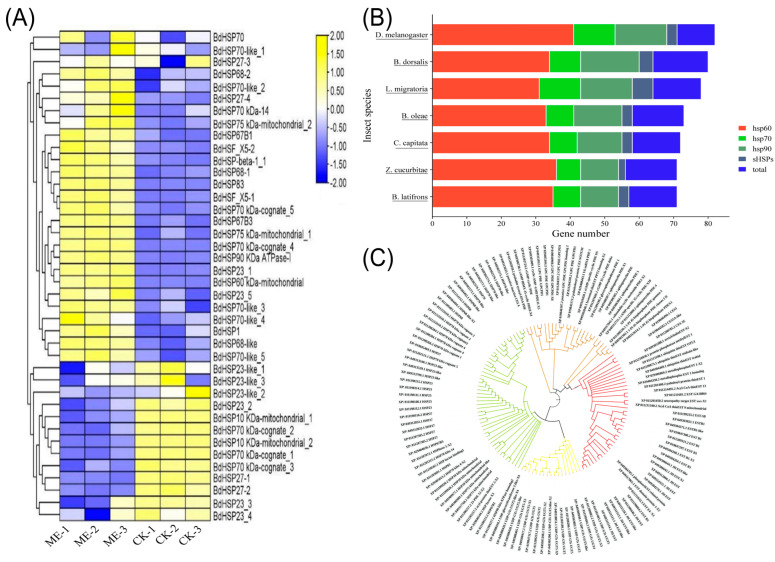
Identification, phylogenetic analysis, and expression profiling of heat shock protein (HSP) genes in *Bactrocera dorsalis*. (**A**) Heatmap and hierarchical clustering illustrating the expression patterns of identified HSP genes. The color scale indicates relative expression levels (yellow: high expression; blue: low expression). (**B**) Comparison of HSP gene family counts (*hsp60*, *hsp70*, *hsp90*, and *sHSPs*) across seven insect species, including *B. dorsalis* and *D. melanogaster*. (**C**) Phylogenetic tree constructed from the amino acid sequences of the identified HSP proteins, with branches colored to distinguish between different HSP subfamilies.

HSPs function as molecular chaperones involved in protein folding and cellular protection under physiological stress and are classified here as stress-response genes rather than detoxification enzymes. ME exposure significantly altered HSP expression with distinct tissue-specific patterns (Figure 7A).

In head tissue of ME-R males, 40 HSP genes were identified, of which 12 were significantly upregulated, and 28 were downregulated (FDR < 0.05). *HSP10* exhibited the most marked upregulation (9.4-fold), suggesting activation of the mitochondrial chaperone system in response to ME-induced metabolic stress. *BdorHSP67B3* was the most strongly downregulated (−4.5-fold). In head tissue of ME-NR males, the same 40 HSP genes were assessed; only 4 were significantly upregulated, with *HSP10* again showing the greatest increase (8.3-fold), while 36 genes were downregulated. *BdorHSP70* (−6.6-fold) and *BdorHSP68-like* (−6.2-fold) showed the most substantial reductions, suggesting broad suppression of cytoplasmic chaperone activity in non-feeding males. In gut tissue, ME exposure altered 28 HSP genes, with 10 upregulated and 18 downregulated. The predominance of HSP downregulation in gut tissue, the primary site of ME metabolism, contrasts with the selective upregulation of *HSP10* in the head, indicating that stress management is managed through organ-specific mechanisms rather than a uniform systemic heat shock response (Table S11). The comparative abundance of HSP gene families across *B. dorsalis* and five other insect species, and the evolutionary relationships among *B. dorsalis* HSP proteins inferred from maximum-likelihood phylogenetic analysis, are presented in (Figure 7B,C).

### 3.6. Validation of DEGs by qRT-PCR

To further verify the reliability of the RNA-seq data, we randomly selected 14 DEGs for qRT-PCR validation. The genes examined included *BdOR94a-like*, *BdOBP83a*, *BdOR33b-like*, *BdOR63ax2*, *BdOR94b*, *BdOBP69a*, *BdOR63ax2*, *BdOBP410_X1*, *BdOR63a*, *BdOR7a-like*, *BdOR13a-X1*, *BdOR83aX1*, *BdOR94a-like2*, and *BdOR83aX2*. The qRT-PCR results were consistent with the RNA-Seq data and showed significant upregulation of these genes in the ME-treated male group (Figure S5). These findings validate the RNA-seq results and further support the reliability and robustness of the transcriptome analysis.

## 4. Discussion

Transcriptomic comparison between-fed and unfed control males of *B. dorsalis* revealed numerous DEGs enriched in KEGG pathways related to xenobiotic and plant secondary compound processing. Key gene families included CYPs, GSTs, UGTs, OBPs, CSPs, multidrug resistance-associated proteins (MRPs), and ATP-binding cassette (ABC) transporters. Our findings also revealed substantial modulation of chemosensory receptor genes, highlighting a coordinated transcriptional response spanning both olfactory detection and metabolic clearance of ME.

Exposure to xenobiotics often induces cellular stress, triggering a protective heat shock response. Critically, HSPs are not known for traditional detoxification genes; rather, they function as molecular chaperones involved in protein folding, stress tolerance, and cellular protection under physiological stress conditions. The predominant downregulation of HSPs, particularly in gut tissues (Section 3.6) (Table S11), suggests that ME imposes a sustained systemic stress rather than an acute heat shock response, with tissue-specific regulation indicating organ-level differences in stress management capacity.

Insect CYPs degrade plant toxins and synthetic insecticides, driving the evolution of resistance [67]. Xenobiotic detoxification typically proceeds in three phases: activation/functionalization (Phase I), conjugation (Phase II), and excretion (Phase III) (Figure S5). Before these enzymatic phases, peripheral proteins such as OBPs and CSPs are thought to facilitate the initial detection, binding, and transport of hydrophobic xenobiotics across the aqueous sensillum lymph toward membrane-bound receptors or detoxifying enzymes [68,69,70]. In the present study, two OBPs and CSPs were significantly upregulated in ME-fed males, suggesting their involvement in the initial recognition, solubilization, and delivery of ME to downstream detoxification machinery.

In Phase I detoxification, CYPs are the primary oxidases responsible for introducing polar groups into lipophilic xenobiotics, thereby increasing their reactivity and water solubility. In *B. dorsalis* males exposed to ME, numerous CYP transcripts were significantly upregulated, including *CYP6a21*, *CYP4p1*, *CYP318a1*, *CYP4p1*-like, *CYP4ad1*, *CYP309a2*-like, *CYP12e1*, *CYP6a2*, *CYP6a13*, *CYP313a4*, *CYP6a9*, *CYP28a5*, *CYP4d1*, *CYP304a1*, *CYP12e1*, CYP6a9-like, *CYP6d5*, *CYP18a1*, *CYP313a4*, *CYP12b1*, *CYP12c1*, *CYP313a4*, *CYP6a14*, CYP12a5, *CYP303a1*, and *CYP12b2* (Table S8). This strong induction indicates that these CYPs are actively involved in the oxidative metabolism of ME, consistent with their established role in phenylpropanoid and insecticide detoxification across insect species. In contrast, few CaE genes showed significant differential expression, such as juvenile hormone (LOC105224146, LOC105224147, LOC105224154, esterase B1, and 1-phosphatidylinositol 4,5-bisphosphate phosphodiesterase gamma-1) (Table S10), and ME exposure did not markedly alter overall CaE activity. These results suggest that, unlike some insecticides that target or are hydrolyzed by CaEs, ME is primarily metabolized through the CYP-mediated pathway in *B. dorsalis* rather than esterase-dependent routes in *B. dorsalis* males.

Overall, the transcriptomic highlights a coordinated detoxification strategy in which chemosensory binding proteins facilitate initial uptake, followed by extensive CYP-dependent oxidation as the major phase I mechanism (Figure 5) for ME metabolism in male *B. dorsalis*. Altogether, the structure of the phylogeny (Figure 6) emphasizes that the ME–activated *BdorOr94a*-like receptor belongs to a rapidly evolving, male-biased chemosensory subfamily, providing an evolutionary framework for understanding how *B. dorsalis* has developed sex-specific sensitivity to plant semiochemicals crucial for its mating system. These findings illuminate the molecular evolution underlying specialist olfactory behaviors and highlight the *OR94* subfamily as a key target for future functional and comparative studies. The most striking finding of the transcriptome data is the significant downregulation of the *Orco*. As *Orco* is essential for the function of most ligand-binding ORs, its downregulation suggests a broad, systemic dampening of the olfactory sensing capability in the antennae. This could indicate a response mechanism to desensitize the fly’s olfactory system to prevent overstimulation by the potent pheromone cue [71].

Furthermore, the expression changes in specific ORs are noteworthy. While most of the rest of the ORs show no significant change, the downregulation of a small set (e.g., *Or49a* and *Or59a*) and the upregulation of two others (putative *Or85d* and *Or7a*) suggest that ME exposure may trigger a specific reconfiguration of the olfactory receptor repertoire, potentially tuning the system’s sensitivity to certain odors. The overall pattern points toward ME having a profound impact on the peripheral olfactory machinery in the head, primarily through the suppression of the essential *Orco* gene.

Ninety-three chemosensory genes were found in the male *B. dorsalis,* considering both genome and transcriptome data (Table S6). Of these genes were annotated in the automatic predictions. Eight of these transcripts were previously identified in the male head and an antennae transcriptome, *BdorOBP2* [58], *BdorOBP56f*-2 [70], *BdorOBP69a* [69], *BdorOBP83b* [57], *BdorOR88a* [23], *BdorOR94b1* [37], BdorOBP83a-2 [72], *BdorOrco* [73], and *BdorOR63a*-1 in antennae [23]. We further investigated our new sequence to allow the identification of formerly unpredicted sequences in the genome (Figure 1 and Figure 6, Tables S5 and S7). It is also worth considering whether the observed changes in detoxification gene expression are entirely attributable to direct ME metabolism or whether ME sensing triggers broader developmental or physiological programs in males. ME ingestion is associated with enhanced mating success and may activate hormonal or reproductive cascades in which CYPs and other enzymes serve secondary roles beyond xenobiotic clearance. This possibility warrants investigation in future studies. In contrast to the OR profile, receptors show a more varied response to ME exposure. The presence of significantly regulated GRs (11 genes) indicates that the gustatory system (likely involving the proboscis and pharyngeal taste organs) is actively responding. The upregulation of bitter taste receptors like “GR for bitter taste *Gr22e*-like” could reflect an aversive response, as bitter taste often signals toxicity. Conversely, the downregulation of other GRs might indicate a suppression of certain taste pathways. A notable observation is that a substantial subset of 15 GR genes showed zero expression change (log_2_FC = 0, FDR = 1), suggesting they are either not expressed in the head or are completely unresponsive under the tested conditions. This highlights the specificity of the response, affecting a particular set of GRs involved in taste perception, which may be crucial for evaluating the palatability or toxicity of xenobiotics upon contact or ingestion.

In conclusion, this study provides clear evidence that ME exposure triggers a comprehensive detoxification and stress response in adult male *B. dorsalis*, characterized by the strong induction of CYPs, UGTs, and specific CaEs, alongside the dynamic modulation of chemosensory receptor genes. The widespread regulation of HSPs further emphasizes the significant physiological impact of ME ingestion. Future functional studies utilizing RNAi or other reverse-genetics approaches are required to definitively validate the roles of these candidate genes in xenobiotic resistance and pheromone sequestration. Inhibiting key upregulated CYPs (particularly *CYP309a2*, *CYP313a4*, and *CYP6a22*) or ME-binding OBPs via synergistic chemical agents or RNAi could potentiate conventional lure-and-kill programs, potentially reducing ME field application rates while maintaining pest suppression efficacy.

## Data Availability

All data generated or analyzed during the study are included in this article (and its Supplementary Information Files). All genome sequence data are available at the GenBank under the Accession numbers (BioProjects PRJNA1102686, PRJNA1102347, PRJNA1102690, and PRJNA1102693). Raw sequence data are available at the NCBI SRA site with the accession numbers SRA: SRR28752167, SRR28745642, SRR28752189, and SRR28752407.

## Supplementary Materials

The following supporting information can be downloaded at: https://www.mdpi.com/article/10.3390/insects17040416/s1.

## Abbreviations

The following abbreviations are used in this manuscript:

ME: methyl eugenol; KEGG: Kyoto Encyclopedia of Genes and Genomes; DEGs: Differentially expressed genes; PCA: Principle component analysis; GO: Gene Ontology; MAPK: Mitogen-activated protein kinase; TF: Transcription factors; ME-T1R1-a: Methyl eugenol treatment 1 row 1; ME-T1R2-a: Methyl eugenol treatment 1 row 2; ME-T1R3-a: Methyl eugenol treatment 1 row 3; CK-1: control-1; CK-2: control-2; CK-3: control-3; A: Head; B: Wings and legs; C: Gut; VC: Volatile compound; Olfactory receptors (ORs); IRs: Ionotropic receptors; DMP: Dimethoxy phenol; (E)-CF (E)- coniferyl alcohol; OSNs: Olfactory sensory neurons.
