# Supplementary material for: Understanding the Chemosensory and Detoxification Mechanisms in the Oriental Fruit Fly, Bactrocera dorsalis
Source: Insects. 2026 Apr 14;17(4):416. doi: 10.3390/insects17040416 (PMC13116743; doi:10.3390/insects17040416)
Supplement: Supplementary file 1 [file insects-17-00416-s001.zip › insects-4124627-supplementary.pdf]

Supplementary Information Tables and Figure

Understanding the chemosensory and detoxification mechanisms in the Oriental fruit fly, *Bactrocera dorsalis*

Saleem Jaffar[1] Yongyue Lu[1]*

[1]Department of Entomology, College of Plant Protection, South China Agricultural University, Guangzhou 510642, China

*Correspondence: luyongyue@scau.edu.cn

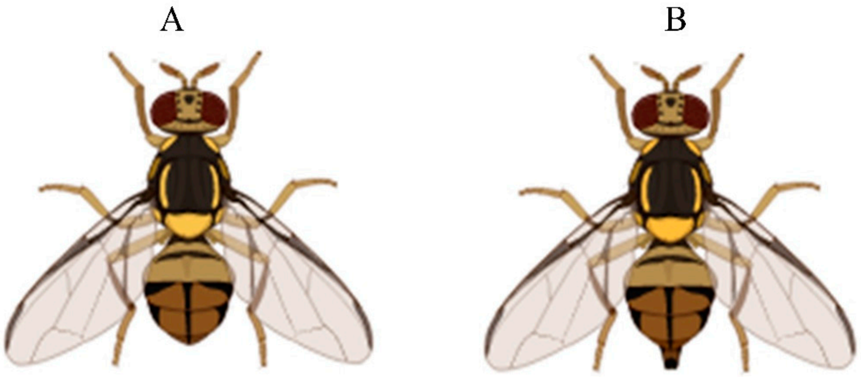

Figure S1. Male (**A**) and female (**B**) adults oriental fruit fly, *Bactrocera dorsalis.* The figures created into Biorender Scientific Image and Illustration Software | BioRender

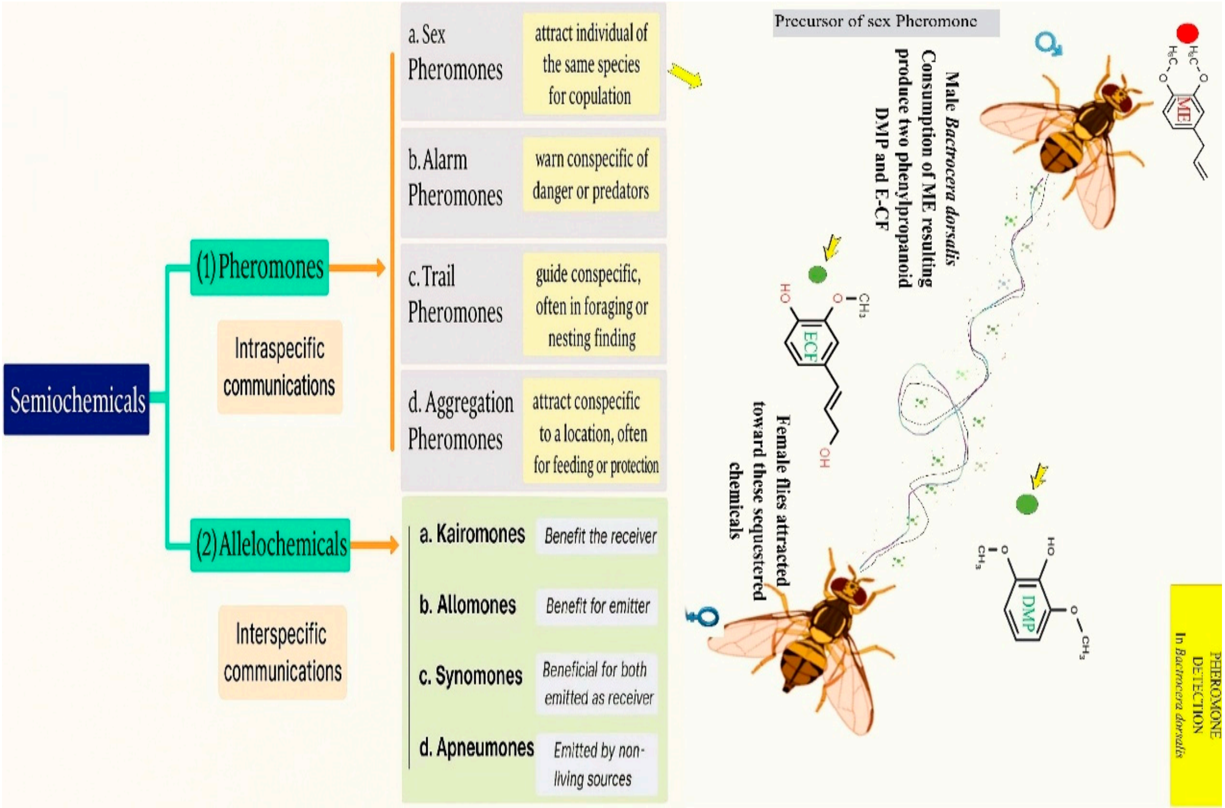

Figure S2. Chemical mediators of ecological interactions: a functional classification of semiochemicals in insects and *Bactrocera dorsalis* to methyl eugenol.

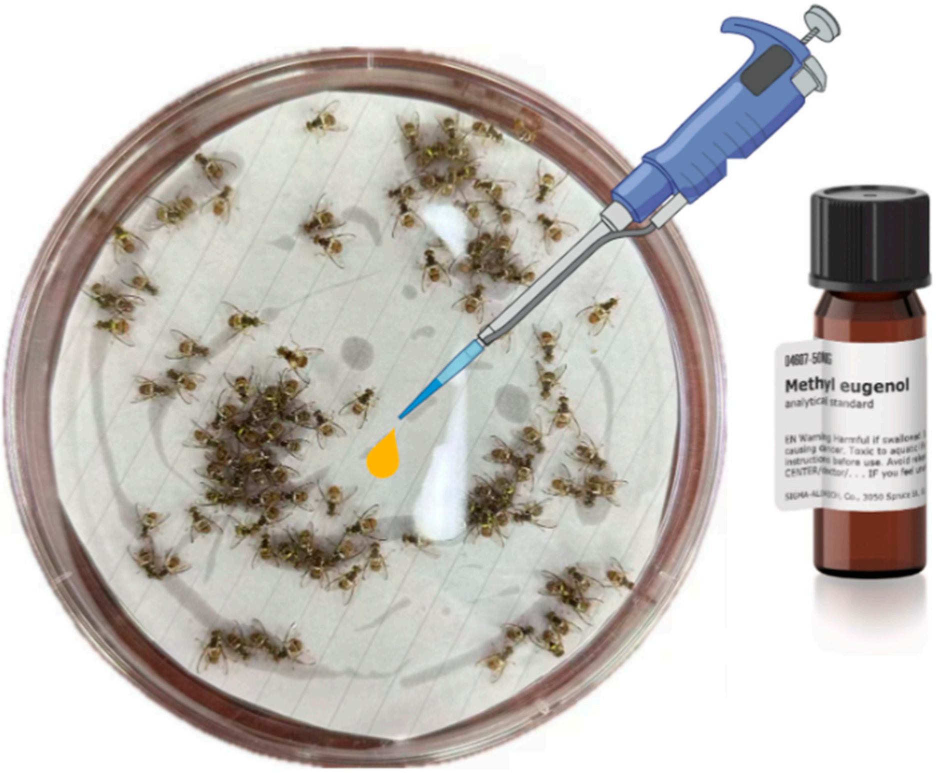

Figure S3. Methyl eugenol application on adult males of *Bactrocera dorsalis*. This figure created in Scientific Image and Illustration Software | BioRender

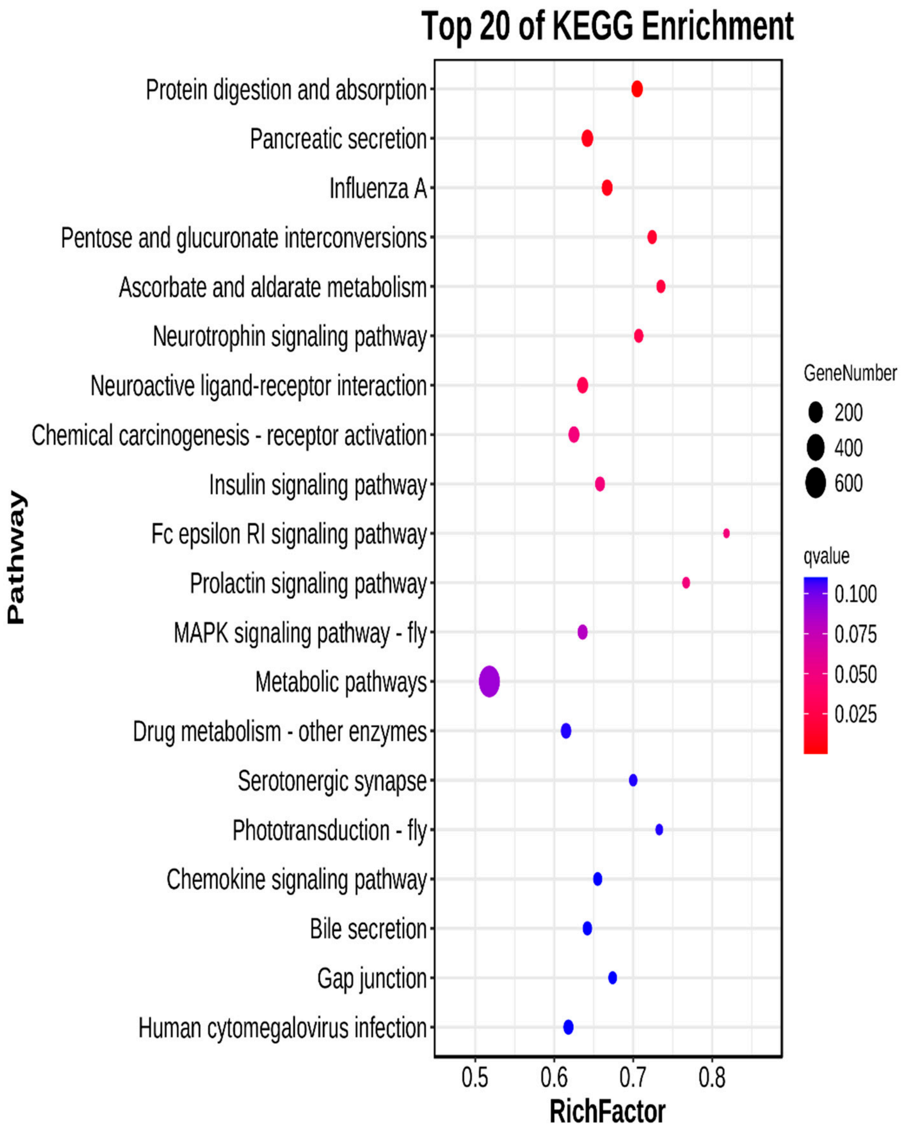

Figure S4. Top 20 KEGG pathway enrichment analysis of differentially expressed genes in male *Bactrocera dorsalis* following methyl eugenol (ME) exposure. Dot plot showing the 20 most significantly enriched KEGG pathways. X-axis: Rich Factor (ratio of DEGs mapped to a pathway relative to all annotated genes in that pathway). Dot size represents gene count; color represents q-value (red: $q < 0.025$; blue: $q \approx 0.100$). Biologically relevant pathways to *B. dorsalis* include metabolic pathways, drug metabolism - other enzymes, MAPK signaling, and neuroactive ligand-receptor interaction. Analysis was performed using the KEGG Automatic Annotation Server with Bonferroni correction ($q \leq 0.05$).

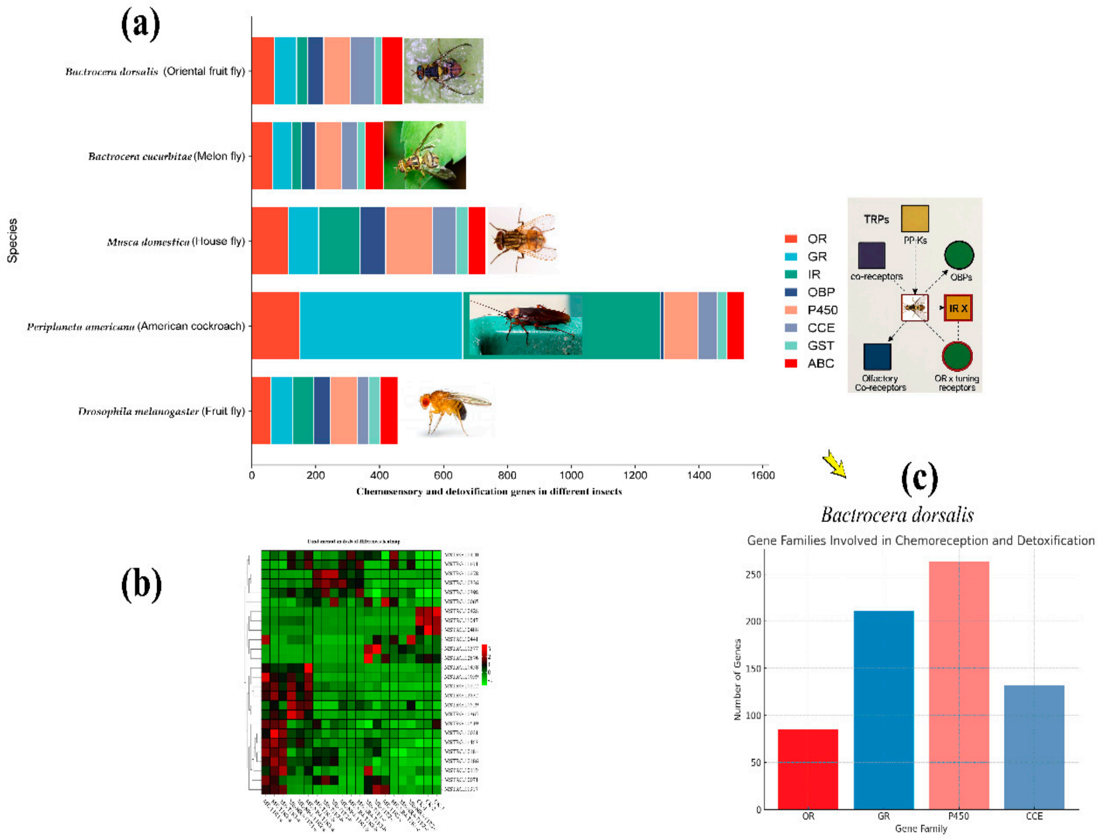

Figure S5. (a) Chemosensory and detoxification genes in agricultural insect pest multicolor bars represents the % of the gene x axis indicates the name of the insects and the y axis represents numbers of genes. (b) Fundamental analysis of differences treatment vs control (c). Gene families show in different tissues head, gut, wing and leg of male *Bactrocera dorsalis* OR 85 genes; GR (gustatory receptors) 211 genes; P450 (cytochrome P450s) 263 genes; CCE (carboxyl/cholinesterase) 132 genes respectively.

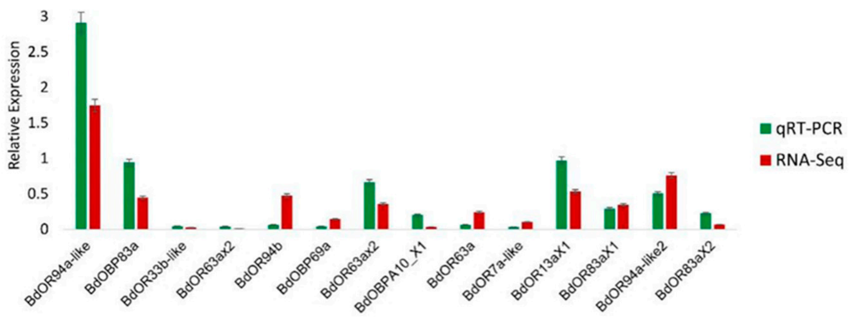

Figure S6. Validation of DEGs via RT-qPCR male *Bactrocera dorsalis* adult. The bar chart compares the relative expression of selected DEGs, with green and red bars representing different samples. The results are generally consistent with RNA-Seq data in both expression levels and direction. *BdorOR94a-like* and *BdorOBP83a* show high expression, reinforcing their likely functional roles in ME detection. These validated genes predominantly relate to odorant detection, supporting the hypothesis that ME exposure influences olfactory gene expression and potentially affects male behavioral responses.

Table. S1. The primers in used in this study for gene expression by qPCR analysis.

Primer name	Nucleotide sequences (Forward) (5′-3′)	Nucleotide sequence (Reverse) (5′-3′)
LOC125778953	TCGCCTATTCGCTTCTCAG	TGGGTTGGCGAGGTAGGAG
LOC125777344	TTGGTTCAAGAATGGGACA	AGGTCATACCCTACCCATC
LOC115066388	TTGAGCACCAATAAGGATG	TTATCGGTCCTATCTCCTC
LOC115066074	ACATTCGGGTAATAGGTCG	ACCATCCGTCATCACCCTA
LOC109579941	TGGCAATAAGGTAACCGAGTC	ACATCCGTAAGAAGTACCAGA
LOC109579324	ATGGTCTGACGCTTTACT	TACAAATAGTAGCACCAC
LOC105232725	GGCTATTCGTTTGCCTTCT	AGTAGGGTTAGTAAGGTGT
LOC105232487	TGGGCAAGAAGTGTTTGTATC	ACCTTACAAGCAGTTGCGATA
LOC105230485	CGCTAACGCAGGTAGAACAT	ACTATTCCGTGACCCTCTTC
LOC105229798	CCGACGCTATTTCAACCG	TTTGCACGGGTTAGGAGG
LOC105226983	ATGTGGAACCCGATGTCTTG	TCGCCACTTATTTATCCACTCG
LOC105226710	AGTGATAAGCCAGCAAAGAAG	GATGGTCTGTCATGTACGGTATG
LOC105226560	AAATGATTACAGCAAGGGAA	TATGTTATCGGTTGCGTGTA
LOC105226556	GTTGAATGCGGAGGAGGT	GCTTGTTACCTACGCTCA
LOC105224894	CTCGGTAACATCGCTTCGTG	CAAACCTGAGGCGGAAGATA
LOC105221865	TTGACCGAAGAACAGAAACA	TTCGTAAACTGAAACGGTAG
**α-tubulin*	CGCATTCATGGTTGATAACG	GGGCACCAAGTTAGTCTGGA

* Note α-tubulin was used as reference control.

Table S2. Statistics of RNA raw sequencing data.

Sample	Raw Data	Clean Data (%)	Adapter (%)	Q20(%)	Q30(%)	GC (%)
ME-T1R1-a	40052488	39946714 (99.74%)	0.06%	97.93%	93.82%	44.83%
ME-T1R2-a	42188832	42064004 (99.70%)	0.03%	97.37%	92.70%	44.10%
ME-T1R3-a	45522896	45341416 (99.60%)	0.06%	97.25%	92.54%	45.82%
ME-NR4-T1R1-a	43780338	43620508 (99.63%)	0.03%	96.84%	91.62%	40.87%
ME-NR4-T1R2-a	40095172	39996356 (99.75%)	0.03%	97.62%	93.21%	40.89%
ME-NR4-T1R3-a	39821110	39713840 (99.73%)	0.04%	97.51%	92.93%	41.28%
ME-T1R1-b	42291824	42168336 (99.71%)	0.04%	97.26%	92.39%	37.18%
ME-T1R2-b	36313552	36225176 (99.76%)	0.08%	97.78%	93.27%	36.23%
ME-T1R3-b	46510972	46344252 (99.64%)	0.05%	96.97%	91.77%	36.78%
ME-NR4-T1R1-b	45429820	45237766 (99.58%)	0.03%	96.49%	90.77%	40.86%
ME-NR4-T1R2-b	42989416	42834506 (99.64%)	0.03%	97.01%	91.87%	40.92%
ME-NR4-T1R3-b	38265916	38166306 (99.74%)	0.02%	97.50%	92.90%	41.24%
ME-T1R1-c	44584134	44459968 (99.72%)	0.04%	97.45%	92.66%	37.83%
ME-T1R2-c	47358788	47224270 (99.72%)	0.03%	97.29%	92.29%	37.89%
ME-T1R3-c	36605538	36519668 (99.77%)	0.04%	97.78%	93.34%	37.78%
ME-NR4-T1R1-c	43871358	43737992 (99.70%)	0.02%	97.13%	92.02%	40.09%
ME-NR4-T1R2-c	37824722	37727976 (99.74%)	0.03%	97.78%	93.48%	40.35%
ME-NR4-T1R3-c	41387610	41301638 (99.79%)	0.05%	97.96%	93.87%	41.11%
CK-1,2,3 head	37001950	36903636 (99.73%)	0.05%	98.13%	94.21%	43.42%
CK-1,2,3 gut	44496478	44298228 (99.55%)	0.05%	97.29%	92.66%	43.52%
CK-1,2,3 leg wing	45978050	45777044 (99.56%)	0.04%	97.05%	92.07%	43.28%

Note: RNA sequencing quality metrics: Q20/Q30 represent the percentages of nucleotides with quality values above 20 and 30, respectively. ME refers to ME; a-head; b-wings and legs; c-gut; CK-control of 16-

day-old virgin male *Bactrocera dorsalis*. High-throughput sequencing of 21 cDNA libraries generated between 36.3 and 47.4 million raw reads per sample, representing three biological replicates each of methyl eugenol-exposed (ME) head (a), wings and legs (b), and gut (c) tissues, ME-NR4-treated counterparts, and age-matched controls (CK). Quality filtering retained over 99.5% of reads across all samples (range: 99.55-99.79%), with minimal adapter contamination (≤0.08%). Sequence quality exceeded standard thresholds, with Q20 scores above 96.49% and Q30 scores above 90.77%, indicating high base-call accuracy. Notable GC content variation was observed between tissue types, ranging from 36.23-37.89% in appendages to 40.09-45.82% in other samples, while control samples showed consistent GC content (43.28-43.52%). These metrics confirm the technical robustness of our transcriptomic data for subsequent differential expression analysis.

Table S3. Summary statistics of transcriptome data of male fly *Bactrocera dorsalis* read mapping. Note: ME, methyl eugenol; a-head; b-wings and legs; c-gut; CK, control of the 16 days old virgin male *Bactrocera dorsalis.*

Sample	Total	Unmapped (%)	Unique_Mapped (%)	Multiple_Mapped (%)	Total_Mapped (%)
**ME-T1-h**	37152930	4626602 (12.45%)	31247886 (84.11%)	1278442 (3.44%)	32526328 (87.55%)
**ME-T1-h**	38502090	5674052 (14.74%)	31477013 (81.75%)	1351025 (3.51%)	32828038 (85.26%)
**ME-T1-h**	41976622	5998838 (14.29%)	34373083 (81.89%)	1604701 (3.82%)	35977784 (85.71%)
**ME-T2-h**	41679956	6250080 (15.00%)	33825404 (81.16%)	1604472 (3.85%)	35429876 (85.00%)
**ME-T2-h**	38359422	5182530 (13.51%)	31774997 (82.83%)	1401895 (3.65%)	33176892 (86.49%)
**ME-T2-h**	38137592	4938007 (12.95%)	31786944 (83.35%)	1412641 (3.70%)	33199585 (87.05%)
**ME-T3-WL**	33534246	4338467 (12.94%)	28067849 (83.70%)	1127930 (3.36%)	29195779 (87.06%)
**ME-T3-WL**	25719638	2705224 (10.52%)	22120220 (86.01%)	894194 (3.48%)	23014414 (89.48%)
**ME- T3-WL**	35314408	4791759 (13.57%)	29329748 (83.05%)	1192901 (3.38%)	30522649 (86.43%)
**ME-T4-WL**	43048442	6232900 (14.48%)	35505860 (82.48%)	1309682 (3.04%)	36815542 (85.52%)
**ME- T4-WL**	40536936	5990760 (14.78%)	33324827 (82.21%)	1221349 (3.01%)	34546176 (85.22%)
**ME- T4-WL**	36644596	4585729 (12.51%)	30972168 (84.52%)	1086699 (2.97%)	32058867 (87.49%)
**ME-T5-g**	40480258	29169665(72.06%)	10449562 (25.81%)	861031 (2.13%)	11310593 (27.94%)
**ME- T5-g**	44874944	39348786(87.69%)	5075818 (11.31%)	450340 (1.00%)	5526158 (12.31%)
**ME- T5-g**	33889686	27974137(82.54%)	5454334 (16.09%)	461215 (1.36%)	5915549 (17.46%)
**ME-T6-g**	42722702	24611211(57.61%)	17317936 (40.54%)	793555 (1.86%)	18111491 (42.39%)
**ME-T6-g**	35768422	15613735(43.65%)	19118099 (53.45%)	1036588 (2.90%)	20154687 (56.35%)
**ME-T6-g**	40302884	17172938(42.61%)	22041372 (54.69%)	1088574 (2.70%)	23129946 (57.39%)
**CK-h**	35604274	7488647 (21.03%)	27056170 (75.99%)	1059457 (2.98%)	28115627 (78.97%)
**CK- g**	42915668	9961312 (23.21%)	31736734 (73.95%)	1217622 (2.84%)	32954356 (76.79%)
**CK-WL**	44246378	10511810(23.76%)	32406025 (73.24%)	1328543 (3.00%)	33734568 (76.24%)

Table S4. Assembly features table for the genome of the *Bactrocera dorsalis*. NCBI BioProject submission, sequence read archive (SRA), accessions numbers and samples

Name	NCBI BioProjects PRJNA	Sub: ID	SRA NO	Link
**ME T-head, W&L**	PRJNA1102690	1102690	SRR28752189	https://www.ncbi.nlm.nih.gov/sra/?term=SRR28752189

**ME T gut**	PRJNA1102693	1102693	SRR28752407	https://www.ncbi.nlm.nih.gov/sra/?term=SRR28752407
**ME-NR4-T1R1-a_1.fq**	PRJNA1102686	1102686	SRR28752167	https://www.ncbi.nlm.nih.gov/sra/?term=SRR28752167
**BD_CK_head-1_1.fq**	PRJNA1102347	1102347	SRR28745642	https://www.ncbi.nlm.nih.gov/sra/?term=SRR28745642

The raw data 'meta samples' (transcriptome) methyl eugenol and control treated on *Bactrocera dorsalis* the data NCBI BioProject submission Abbreviations noted as ME- (methyl eugenol), NR (not responding), T (treatment), a (head) BD (*Bactrocera dorsalis*), CK (control).

*Bd[1] (Jiang, Liang, Wang, & Zhu, 2022), Bd[2] (Yang et al., 2023), Dm[3], Bc[4] (Sim & Geib, 2017), Md[5](Scott et al., 2014), [6]*Pa* (Li et al., 2018),

Table. S5. Expression pattern of odorant receptors in response to ME in male *B. dorsalis* head transcriptome. 86 genes were identified in male *B. dorsalis* head treated with ME and control.

1) LOC105226560 (BdorOR83a)
2) LOC109579413 (odorant receptor 7a)
3) LOC109579261 (BdorOR35a, OR13a.2, OR35a)
4) LOC125775341 (Or63a-like)
5) LOC125776165 (Or83a-like)
6) LOC105224267 (Or63a)
7) LOC125777287 (Or88a-like)
8) LOC125775339 (Or43b-like)
9) LOC105224108 (Or67c-like)
10) LOC105229372 (Or33b-like)
11) LOC125779969 (Or33b-like)
12) LOC105229925 (Or82a)
13) LOC105230030 (BdorOR7a-6)
14) LOC105230485 (BdorOR7a-2, OR42a, OR7a-2)
15) LOC105232725 (BdorGR63a, GR63a)
16) LOC105226510 (OR67d.3)
17) LOC109579410 (Or43b, OR7a-3)
18) LOC109579324 (Or7a-like)
19) LOC105232464 (OR45a)
20) LOC105226810 (Or43b-like)
21) LOC125776739 (GR and Or22-like)
22) LOC125779951 (Or33b-like)
23) LOC105224201 (Or46a)
24) LOC105230726 (BdorOR47b, OR47b)
25) LOC105222886 (BdorOr7a)
26) LOC105231419 (BdorOr30a; OR43a.2; OR49b-2)
27) LOC105223438 (BdorOr59a-like)
28) LOC115066388 (BdorOr33b-like)
29) LOC105232813 (BdorOR43b; OR7a.4)
30) LOC105226556 (Or coreceptor; ORCO; Or83b)
31) LOC105232447 (BdorOR49a; BdorOR49a)
32) LOC105226710 (GR and BdorOr21a-like; GR21a)
33) LOC125778525 (OR7a.1; BdorOR7a-9; Or7a-like)
34) LOC125778953 (BdorOr94a-like)
35) LOC105230489 (BdorOr85c-like)
36) LOC115065780 (BdorOr67d; OR67d.2)
37) LOC115066237 (Or88a; OR88a; BdorOR88a)
38) LOC109579891 (putative Or69a; BdorOR69a.2)
39) LOC115065781 (BdorOr67d-like)
40) LOC105230488 (BdorOr85c-like)
41) LOC125778952 (BdorOr94a-like)
42) LOC125776742 (GR and BdorOr22-like)
43) LOC105224107 (BdorOr67c; OR67c; OR67c.1)
44) LOC125778528 (Or7a-like)
45) LOC105225171 (BdorOr63a; Also known as OR63a.1)
46) LOC105226983 (Or13a; OR13a; OR13a.1; BdorOR13a)
47) LOC115065782 (BdorOr67d; OR67d.1)
48) LOC105233676 (BdorOr43a; OR43a-1; OR43a.1)
49) LOC109579941 (BdorOr94b; OR94b; OR94b.1)
50) LOC105227706 (BdorOr94a-like)
51) LOC105230346 (putative Or69a; BdorOR69a; OR69a.1)
52) LOC105226603 (BdorOr83a)
53) LOC115066594 (BdorOr33b; OR59A)
54) LOC109579790 (BdorOr63a; OR63a-2; OR63a.3)
55) LOC125777344 (BdorOr94a-like)
56) LOC105228210 (BdorOr10a; OR10a; BdorOR10a)
57) LOC109579485 (BdorOr49b; OR49b; OR49b-1)
58) LOC125777064 (Or59a-like; Also known as OR59a)
59) LOC115066182 (Or83a-like)
60) LOC125777286 (BdorOr88a-like; OR88a)
61) LOC115065926 (Or63a-like)
62) LOC105222497 (putative BdorOr85e)
63) LOC105222171 (Or59a; OR59a.1; BdorOR59a)
64) LOC105223483 (BdorOr24a)
65) LOC105230396 (BdorOr7a-like)
66) LOC105225172 (BdorOr63a-like)
67) LOC105232463 (BdorOr45a)
68) LOC105231924 (BdorOr94a)
69) LOC115066074 (BdorOr63a)
70) LOC105222669 (BdorOr2a; OR19a)
71) LOC125775317 (BdorOr67d-like)
72) LOC125776166 (BdorOr83a-like)
73) LOC125779060 (BdorOr63a-like)
74) LOC125776254 (putative BdorOr85e)
75) LOC105226509 (BdorOr67d; OR67d; OR67d.4)
76) LOC105230490 (putative BdorOr85d)
77) LOC105223253 (Or7a; Also known as BdorOR7A)
78) LOC105230821 (BdorOr22c; OR22C)
79) LOC105229979 (BdorOr92a; OR92A; BdorOR92a)
80) LOC125780083 (BdorOr59a-like; Also known as OR59a.2)
81) LOC125779055 (BdorOr63a-like)
82) LOC109579893 (BdorOrco2)
83) LOC105227427 (BdorOr63a)
84) LOC105231103 (BdorOr7a; OR7a.6; BdorOR42b)
85) LOC105229921 (Or7a; OR7a-1; OR7a.8; BdorOR7a-1)
86) LOC125778524 (Or43b-like; BdorOR7a.2)

Table. S6. Expression pattern of odorant receptors in response to ME in male *B. dorsalis* head transcriptome.

Symbol	log2(fc)	P Value	FDR	Description
LOC105226556	-4.05239	2.24E-22	1.12E-21	ORCO
LOC105232447	-5.13277	2.21E-08	5.64E-08	Or49a
LOC105223438	-3.3569	5.11E-07	1.19E-06	Or59a-like
LOC125778953	-7.30986	7.36E-07	1.69E-06	Or94a-like
LOC105230490	4.516576	0.000134	0.000263	putative Or85d
LOC115066388	-3.04591	0.000641	0.001184	Or33b-like
LOC105226710	-3.28848	0.001004	0.001821	GR and Or21a-like
LOC105224201	-7.89885	0.001019	0.001847	Or46a
LOC105229979	2.134859	0.002544	0.004439	putative Or92a
LOC105223253	6.47032	0.005592	0.009419	Or7a
LOC115066074	0.63663	0.007221	0.011985	Or63a
LOC105230489	-4.4989	0.007674	0.0127	Or85c-like
LOC105232813	-8.53398	0.0136	0.021841	Or7a
LOC125777064	-4.95842	0.023457	0.036376	Or59a-like
LOC125780083	2.06413	0.027723	0.042658	Or59a-like
LOC105230485	-3.55254	0.034614	0.052505	Or7a
LOC125779055	1.404984	0.04083	0.061323	Or63a-like
LOC125778528	-4.97154	0.052112	0.076979	Or7a-like
LOC105227706	-3.50356	0.072115	0.103975	Or94a-like
LOC105224107	-4.13286	0.076838	0.110241	Or67c
LOC105232725	-1.86506	0.080896	0.11561	GR and Or63a
LOC105222886	-3.59418	0.092869	0.131449	Or7a
LOC105230726	-4.11177	0.1089	0.151992	Or47b
LOC105229921	2.327262	0.110623	0.154205	Or7a
LOC105224108	-4.078	0.112869	0.157009	Or67c-like
LOC125778525	-3.99616	0.126742	0.174678	Or7a-like
LOC105229925	-3.97199	0.130773	0.179708	Or82a
LOC109579891	-7.54689	0.163341	0.219898	putative Or69a
LOC105222669	0.827578	0.167084	0.224482	Or2a
LOC105231419	-7.63178	0.167977	0.225577	Or30a
LOC125778952	-3.54553	0.17094	0.229221	Or94a-like
LOC105231103	0.447075	0.173138	0.231892	Or7a
LOC105228210	-3.28421	0.174753	0.233871	Or10a
LOC105230821	5.763766	0.189868	0.2519	Or22c
LOC105230030	-2.51177	0.198755	0.262216	Or7a-like
LOC125778524	2.018536	0.205089	0.269769	Or43b-like
LOC115066182	-3.81616	0.238888	0.309386	Or83a-like
LOC109579324	-2.04419	0.24034	0.310991	Or7a-like
LOC109579485	-7.21917	0.251986	0.324283	Or49b
LOC105224267	-7.51043	0.264391	0.338106	Or63a
LOC105230346	-3.4011	0.27953	0.355013	putative Or69a
LOC109579893	1.584963	0.314667	0.393959	OrOr2
LOC109579790	-6.96193	0.314902	0.394119	Or63a
LOC115065782	-2.29257	0.32369	0.403632	Or67d
LOC105229372	-1.83732	0.325253	0.405444	Or33b-like
LOC105233676	-2.5784	0.340966	0.422689	Or43a
LOC105227427	1.016583	0.356747	0.439755	Or63a
LOC125779969	-2.15285	0.37755	0.461432	Or33b-like
LOC125777344	-6.76818	0.379469	0.463429	Or94a-like
LOC109579941	-1.87074	0.446531	0.533961	Or94b
LOC125776739	-1.65786	0.490093	0.576392	GR and Or22-like
LOC125776166	-6.97346	0.513843	0.60027	Or83a-like
LOC125777286	-2.29842	0.534597	0.619567	Or88a-like
LOC105226560	-1.22574	0.542579	0.627475	Or83a
LOC105225171	-6.27302	0.561808	0.6469	Or63a
LOC125776742	-2.12217	0.608228	0.691405	GR and Or22-like
LOC115065781	-5.93074	0.610809	0.692119	Or67d-like
LOC105232464	-6.07325	0.612813	0.692119	Or45a
LOC105226603	3.369234	0.617577	0.692119	Or83a
LOC105231924	2.321928	0.617577	0.692119	Or94a
LOC109579410	-1.8909	0.61991	0.694077	Or43b
LOC115066594	-0.07215	0.637925	0.710873	Or33b
LOC115065780	-1.87447	0.662402	0.732791	Or67d
LOC125775317	-2.03157	0.674592	0.743627	Or67d-like
LOC105222171	-0.40788	0.681279	0.74976	Or59a
LOC115065926	-6.23522	0.725425	0.788861	Or63a-like
LOC125775341	-5.70044	0.73103	0.791588	Or63a-like
LOC125776165	-5.96963	0.73103	0.791588	Or83a-like
LOC125776254	-0.21681	0.735755	0.795909	putative Or85e
LOC115066237	-1.5959	0.742405	0.800739	Or88a
LOC125779951	-1.39375	0.756181	0.813822	Or33b-like
LOC109579413	-1.51221	0.829298	0.877597	Or7a
LOC105226510	-0.75081	0.840237	0.886141	Or67d
LOC105226810	-4.80735	0.847299	0.886805	Or43b-like
LOC125777287	-4.9542	0.847518	0.886805	Or88a-like
LOC105226983	-1.00228	0.854862	0.891238	Or13a
LOC105226509	-1.38904	0.890865	0.921216	Or67d
LOC105222497	-1.08746	0.962032	0.98108	putative Or85e
LOC125775339	-1.10975	0.975838	0.981312	Or43b-like
LOC105230488	-4.16993	0.977756	0.981312	Or85c-like
LOC105223483	-4.45943	0.977756	0.981312	Or24a
LOC105225172	-4.11548	0.977756	0.981312	Or63a-like
LOC105230396	-3.36923	0.977756	0.981312	Or7a-like
LOC105232463	-4.64386	0.977756	0.981312	Or45a
LOC125779060	-4.82443	0.977756	0.981312	Or63a-like
LOC109579261	-1.1366	0.994574	0.995459	Or35a

Table S7. Expression pattern of GR receptors in response to ME in male *B. dorsalis* head transcriptome.

Symbol	log2(fc)	P Value	FDR	Description
ncbi_105225227	4.509589765	6.05483E-25	3.3713E-24	GRs8a
ncbi_105231250	4.92702264	1.51196E-07	3.64521E-07	GRs for bitter taste 22e-like
ncbi_105223121	0.420150228	1.00264E-06	2.29175E-06	putative GRs28b
ncbi_105231253	8.207827794	0.00148602	0.002654294	GRs for bitter taste 22e-like
ncbi_105230597	2.668245633	0.008741156	0.014354652	putative GRs2a
ncbi_105223070	1.615287038	0.012355105	0.019951217	putative GRs28a
ncbi_115066260	-3.886132035	0.014931077	0.023834647	putative GRs98b
MSTRG.3305	0.870057759	0.015483196	0.024643385	GRs2, partial [*Athetis dissimilis*]
ncbi_105229190	2.444099965	0.025804086	0.039851796	putative GRs59f
ncbi_105231252	6.882643049	0.038252697	0.057647042	GRs for bitter taste 22e-like
ncbi_115066509	2.791413378	0.096852411	0.136585057	putative GRs22d
ncbi_105233098	2.49683159	0.10217986	0.143457944	GRs5a for trehalose
ncbi_105225214	0.370267629	0.130495639	0.17939471	GR8a
ncbi_109579940	0.9231064	0.152009442	0.206160603	putative GR94a
ncbi_105222715	0.45691838	0.170868667	0.229150485	putative GR58a
ncbi_105230819	5.48112669	0.226378702	0.29454201	GR68a
ncbi_105224551	1.153805336	0.350747987	0.433493097	GRs for sugar taste 43a
ncbi_115066667	0.613390909	0.369683468	0.453675651	GRs for sugar taste 64f
ncbi_105225721	-0.076757199	0.390478666	0.475295195	GR for bitter taste 66a-like
ncbi_105228990	0.215728691	0.445692298	0.533045073	putative GR 47b
ncbi_105228212	-6.658211483	0.457149145	0.544666809	putative GR59d
ncbi_115066305	-6.386581053	0.464127576	0.551396528	putative GR22c
ncbi_105223308	4.857980995	0.474428927	0.561309708	putative GR39b
ncbi_105225869	5.273018494	0.474428927	0.561309708	putative GR89a
ncbi_125779736	5.26052755	0.479826105	0.56591005	putative GR22a
ncbi_115066029	-7.055282436	0.513842931	0.600269991	GR for sugar taste 43a-like
ncbi_105227650	-2.686500527	0.534903886	0.619873169	putative GR98b
ncbi_125778345	0.835613182	0.569138257	0.654155688	putative GR39b
ncbi_105233089	-0.258828295	0.604633275	0.687872047	GR for sugar taste 64e
ncbi_105233094	-5.48112669	0.608306084	0.691405167	GR for sugar taste 64a
ncbi_125778229	-6.345774837	0.613515389	0.692118859	putative GR39b
ncbi_105228213	4.321928095	0.617577426	0.692118859	putative GR22a
MSTRG.3804	-0.478047297	0.676038229	0.744721432	putative GR28b [*Bactrocera tryoni*]
ncbi_105224525	-0.26598169	0.730289695	0.791400654	GR for sugar taste 43a
ncbi_125778996	-5.781359714	0.736325762	0.795908729	putative GR77a
ncbi_105228623	-2.030373649	0.743193178	0.801436642	GR23a
ncbi_105233095	-2.08912589	0.766741868	0.823008241	GR for sugar taste 64b
ncbi_105225752	0.080170349	0.779693214	0.834827072	putative GR93c
ncbi_105233096	-5.285402219	0.844364058	0.886141235	GR for sugar taste 64c-like
ncbi_109579632	-5.183221824	0.844364058	0.886141235	putative GR98b
ncbi_105228631	-5.285402219	0.850478229	0.887363225	putative GR39b
ncbi_125777486	-5.029747343	0.850478229	0.887363225	putative GR36c
MSTRG.3805	-0.876470082	0.8892941	0.920247952	putative GR28b [*Bactrocera tryoni*]
ncbi_105229094	-0.996776099	0.937144205	0.960791216	GR for bitter taste 66a
ncbi_115066303	-4.604862058	0.977756124	0.981311845	putative GR59b
ncbi_125779002	-4.857980995	0.977756124	0.981311845	GR for sugar taste 64f-like
ncbi_105233097	-4.36923381	0.977756124	0.981311845	GR5a for trehalose-like

**Table continues**				
**ncbi_125777135**	-4.196397213	0.977756124	0.981311845	putative GR28b
**ncbi_125779727**	-3.772589504	0.977756124	0.981311845	putative GR22b
**ncbi_115066510**	-1.158429363	0.999771547	0.999771547	putative GR22b
**ncbi_105225480**	0	1	1	putative GR57a
**ncbi_105225751**	0	1	1	GR for bitter taste 93a
**ncbi_105228209**	0	1	1	GR10a
**ncbi_105228211**	0	1	1	putative GR36b
**ncbi_105228625**	0	1	1	GR23a-like
**ncbi_105228627**	0	1	1	putative GR2a
**ncbi_105228629**	0	1	1	putative GR39b
**ncbi_105228630**	0	1	1	putative GR39b
**ncbi_105231251**	0	1	1	GR for bitter taste 22e-like
**ncbi_125778227**	0	1	1	putative GR39b
**ncbi_125778228**	0	1	1	putative GR39b
**ncbi_125779000**	0	1	1	putative GR77a
**ncbi_125779953**	0	1	1	putative GR98b

Table S8. Expression pattern of CYPs in ME non-responsive male *B. dorsalis* gut

Symbol	log2(fc)	P Value	FDR	Description
**LOC105234261**	8.584313936	8.4093E-293	3.0011E-289	CYP450 4g1
**LOC105234038**	2.668595792	7.87148E-97	7.39246E-95	probable CYP450 28d1
**LOC105222603**	2.135527863	1.17764E-95	1.08457E-93	CYP450 6g1-like
**LOC105231049**	8.58435492	2.02675E-94	1.81961E-92	probable CYP450 304a1
**LOC125777717**	3.658461136	4.32274E-85	3.10086E-83	CYP450 6g1-like
**LOC105230212**	2.668973531	2.50076E-79	1.50626E-77	CYP450 6a2
**LOC105224875**	4.532454584	2.13753E-72	1.00704E-70	CYP450 302a1, mitochondrial
**LOC105231454**	6.820823775	2.28151E-64	8.755E-63	CYP450 4d8
**LOC125777726**	-3.068335773	2.86886E-51	7.40561E-50	CYP450 4p1-like
**LOC105230949**	1.753362902	1.68379E-48	3.92748E-47	probable CYP450 6a14
**LOC105232220**	1.092901906	6.02281E-45	1.24965E-43	CYP450 4ae1
**LOC125775783**	6.684709079	2.196E-44	4.45916E-43	probable CYP450 310a1
**LOC105231113**	-2.727004717	2.16387E-38	3.571E-37	probable CYP450 12e1, mitochondrial
**LOC105227637**	2.18738904	3.31579E-36	5.07319E-35	probable CYP450 12a5, mitochondrial
**LOC105228765**	8.588185784	1.34741E-35	2.02253E-34	probable CYP450 4ac1
**LOC105224483**	2.951676524	3.30907E-34	4.67692E-33	probable CYP450 28d1
**LOC105228198**	-2.174941657	1.55823E-29	1.82476E-28	CYP450 6a9-like
**LOC105226034**	-2.486127492	2.6459E-29	3.06328E-28	probable CYP450 6d4
**LOC125775333**	7.280541638	3.42968E-27	3.5999E-26	CYP450 4g1-like
**LOC105225364**	-2.030747027	1.27986E-26	1.31061E-25	CYP450 9b2
**LOC105228038**	1.572629281	2.70283E-23	2.34404E-22	probable CYP450 6a14
**LOC125775326**	14.66094296	2.54795E-22	2.10975E-21	probable CYP450 313a4
**LOC105230837**	14.57398242	4.91928E-22	4.03811E-21	probable CYP450 313a4
**LOC105226935**	1.032815868	6.59246E-22	5.37449E-21	probable CYP450 6v1
**LOC105233810**	1.649889388	4.40418E-19	3.10008E-18	probable CYP450 6g2
**LOC125776206**	-2.622937669	4.83155E-18	3.19307E-17	probable CYP450 6a21
**LOC105233910**	3.468553009	3.75396E-17	2.35137E-16	CYP450 4c3
**LOC105231082**	1.033784276	4.22693E-16	2.47496E-15	probable CYP450 12e1, mitochondrial
**LOC105234040**	12.64348044	4.85228E-16	2.83182E-15	probable CYP450 309a1
**LOC105222980**	1.568625218	5.7578E-16	3.3466E-15	probable CYP450 4s3
**LOC105225824**	0.17451098	1.18367E-15	6.75334E-15	probable CYP450 9f2
**LOC125778720**	-3.650474983	1.18884E-15	6.78012E-15	probable CYP450 6a13
**LOC105230713**	-2.077786351	5.40844E-15	2.96033E-14	probable CYP450 301a1, mitochondrial
**LOC105233213**	-2.887588058	1.1318E-14	6.05562E-14	probable CYP450 49a1
**LOC125778705**	1.968677738	2.89952E-14	1.51448E-13	probable CYP450 305a1
**LOC105230201**	-1.618924218	5.82516E-14	2.96133E-13	CYP450 6a8-like
**LOC125777716**	4.729573801	1.05899E-13	5.27645E-13	probable CYP450 6t3
**LOC105224048**	-2.004222962	1.23559E-13	6.12645E-13	CYP450 6a2
**LOC105222597**	7.040782866	3.12118E-13	1.49815E-12	probable CYP450 6g2
**LOC105222668**	4.928734161	3.94405E-13	1.88047E-12	probable CYP450 308a1
**LOC105222492**	1.536730775	6.29871E-13	2.96257E-12	probable CYP450 313b1
**LOC105228037**	-4.845331243	1.94223E-12	8.78774E-12	probable CYP450 6a23

**LOC125777727**	-1.785176422	2.96376E-12	1.32046E-11	CYP450 4p1-like
**LOC105224323**	0.590816435	4.71962E-12	2.07492E-11	CYP450 4g15
**LOC125778516**	1.48787279	4.87708E-12	2.14019E-11	probable CYP450 4s3
**LOC105229174**	-1.471333292	6.65937E-12	2.8956E-11	CYP450 4p1
**LOC105230836**	11.41662162	1.53502E-11	6.50994E-11	probable CYP450 313a4
**LOC105228199**	-2.168076785	2.49656E-10	9.71602E-10	CYP450 6a9
**LOC105227636**	2.05632997	1.29452E-09	4.76023E-09	CYP450 CYP12A2
**LOC105224383**	7.113568546	1.77568E-09	6.45806E-09	probable CYP450 309a2
**LOC105230199**	0.189949137	3.39674E-09	1.20919E-08	probable CYP450 317a1
**LOC125777714**	7.034693166	3.43729E-09	1.22332E-08	probable CYP450 6t3
**LOC105232008**	1.331673876	3.64034E-09	1.29204E-08	CYP450 4d2
**LOC125777030**	-4.559525803	1.07291E-08	3.67463E-08	probable CYP450 6a23
**LOC105230839**	10.00515667	3.15047E-08	1.03768E-07	probable CYP450 313a4
**LOC105234048**	-1.21698571	7.20051E-08	2.30155E-07	NADPH--CYP450 reductase
**LOC105227668**	1.653564581	1.19431E-07	3.73714E-07	probable CYP450 4aa1
**LOC125776302**	-1.589313599	1.74769E-07	5.38608E-07	probable CYP450 6d4
**LOC125778964**	-4.136171395	1.13319E-06	3.26005E-06	probable CYP450 4d14
**LOC112694761**	0.071230848	1.15067E-06	3.30899E-06	CYP450 Cyp309a1-like
**LOC105233823**	-2.084417971	1.71682E-06	4.8501E-06	CYP450 6g1-like
**LOC125777718**	0.382283255	2.47645E-06	6.89377E-06	CYP450 6g1-like
**LOC105225301**	3.037302981	2.57357E-06	7.15717E-06	CYP450 307a1
**LOC105227639**	9.116343961	2.7512E-06	7.62887E-06	probable CYP450 12a4, mitochondrial
**LOC125777240**	0.330862339	4.99348E-06	1.35208E-05	probable CYP450 6a23
**LOC105233159**	9.312126628	7.85708E-06	2.08012E-05	probable CYP450 6t1
**LOC125778963**	-2.496747233	9.16789E-06	2.40529E-05	probable CYP450 4d14
**LOC105230935**	-1.547983472	2.36577E-05	5.9309E-05	probable CYP450 6d5
**LOC105232707**	3.711595939	3.11615E-05	7.70403E-05	CYP450 315a1, mitochondrial
**LOC105228214**	-2.254405724	9.44325E-05	0.000221678	CYP450 4d1
**LOC105230198**	0.051595399	0.000188371	0.00042581	probable CYP450 6a13
**LOC105224046**	2.659472778	0.000444683	0.000962232	probable CYP450 6a13
**LOC105224045**	0.178516397	0.00114849	0.002367124	CYP450 6a22
**LOC105228875**	-0.166087214	0.001505847	0.003055575	probable CYP450 6u1
**LOC105224047**	-0.170400139	0.001658616	0.003342757	CYP450 6a22
**LOC105227987**	-2.783493202	0.002488727	0.004914453	probable CYP450 313a4
**LOC105226035**	-2.305049149	0.002561036	0.005049557	probable CYP450 6d4
**LOC125775255**	3.495410916	0.002942619	0.005745611	probable CYP450 12e1, mitochondrial
**LOC125777440**	-0.112874867	0.004166383	0.007944846	probable CYP450 12b2, mitochondrial
**LOC105232227**	0.242895335	0.004327098	0.008226038	probable CYP450 311a1
**LOC105231537**	0.074804217	0.005535842	0.010350248	CYP450 6g1
**LOC125776307**	7.781359714	0.008921598	0.016145514	probable CYP450 313a4
**LOC105231489**	2.500308627	0.011844888	0.021027954	probable CYP450 316a1
**LOC105230202**	-0.168708686	0.012645924	0.022344419	probable CYP450 6a21
**LOC105229193**	0.96051171	0.016502386	0.02865834	CYP450 4p1
**LOC105223016**	0.312861562	0.028284826	0.047146882	probable CYP450 318a1

**LOC115066377**	-1.954135633	0.043280732	0.0699697	CYP450 4p1-like
**LOC105228053**	-1.561737319	0.044285034	0.071463809	probable CYP450 4ad1
**LOC105234039**	1.20378111	0.044522609	0.071814717	CYP450 309a2-like
**LOC125776208**	0.079658675	0.046215232	0.074309803	probable CYP450 12e1, mitochondrial
**LOC105227474**	-0.901146494	0.051120308	0.081435375	CYP450 6a2
**LOC105224050**	-3.868308133	0.072471885	0.112327487	probable CYP450 6a13
**LOC105230834**	6.705056346	0.103985805	0.155727798	probable CYP450 313a4
**LOC105230200**	-0.445553076	0.106497809	0.159122485	CYP450 6a9
**LOC125776207**	-0.89051018	0.119183642	0.175505105	probable CYP450 12e1, mitochondrial
**LOC105228328**	0.290786851	0.157224337	0.225158247	probable CYP450 28a5
**LOC105232013**	0.086117671	0.200353062	0.279437222	CYP450 4d1
**LOC105231048**	6.327926836	0.240512536	0.328107459	probable CYP450 304a1
**LOC105231112**	-0.801282801	0.265508438	0.358540625	probable CYP450 12e1, mitochondrial
**LOC125777239**	-1.224111497	0.276870627	0.371555471	CYP450 6a9-like
**LOC125776596**	0.774118593	0.361673339	0.466343321	probable CYP450 6d5
**LOC105224924**	0.666576266	0.363223033	0.467834413	CYP450 18a1
**LOC105230977**	4.938599455	0.473537662	0.582235153	probable CYP450 313a4
**LOC105226219**	-0.792260372	0.483506585	0.591433735	CYP450 12b1, mitochondrial
**LOC105226210**	-0.495409116	0.511839296	0.618147712	probable CYP450 12c1, mitochondrial
**LOC105228261**	-1.499018092	0.597539505	0.697145283	probable CYP450 313a4
**LOC105228039**	0.836501268	0.612224657	0.711049302	probable CYP450 6a14
**LOC105230203**	-0.533487529	0.68803292	0.768344254	CYP450 6a9
**LOC105227638**	3.736965594	0.818562747	0.857235948	probable CYP450 12a5, mitochondrial
**LOC105229279**	-0.261045853	0.836029057	0.872327665	probable CYP450 303a1
**LOC105226220**	-0.518467089	0.92048043	0.941587828	probable CYP450 12b2, mitochondrial

Table S9. Expression pattern of UGTs in response to ME in male *B. dorsalis* gut transcriptome

Symbol	log2(fc)	P Value	FDR	Description
**LOC105232213**	2.580752187	2.6138E-202	1.528E-200	UDP-glycosyltransferase UGT5
**LOC105224876**	3.023045793	1.32203E-93	2.49244E-92	UDP-glycosyltransferase UGT5
**LOC109579828**	1.937506406	1.41845E-65	1.79762E-64	UDP-glycosyltransferase UGT5
**LOC105231227**	1.976975422	4.00354E-63	4.82088E-62	UDP-glycosyltransferase UGT5
**LOC105231237**	3.868980608	7.19358E-26	3.70752E-25	UDP-glycosyltransferase UGT5
**LOC105230262**	0.607523485	9.03428E-24	4.37598E-23	UDP-glycosyltransferase UGT5
**LOC105226110**	7.236797374	1.69897E-19	6.98373E-19	UDP-glycosyltransferase UGT5
**LOC125777457**	4.961420001	4.34393E-16	1.55004E-15	UDP-glycosyltransferase UGT5-like
**LOC105230659**	0.420167058	1.26219E-13	4.0458E-13	UDP-glycosyltransferase UGT5
**LOC105230266**	1.385269309	1.26544E-13	4.05445E-13	UDP-glycosyltransferase UGT5
**LOC125777875**	1.663658679	1.4523E-13	4.63777E-13	UDP-glycosyltransferase UGT5-like
**LOC125776552**	1.092599888	1.94923E-13	6.19736E-13	UDP-glycosyltransferase UGT5-like
**LOC105223455**	0.342939316	2.26863E-11	6.49627E-11	UDP-glycosyltransferase UGT4
**LOC125777462**	4.611468849	3.81027E-11	1.07704E-10	UDP-glycosyltransferase UGT5-like
**LOC115066621**	1.561941438	2.38275E-05	4.8234E-05	UDP-glycosyltransferase UGT5-like
**LOC105229550**	-0.349475551	0.001171073	0.002076847	UDP-glycosyltransferase UGT5
**LOC105230804**	0.243407936	0.011483349	0.018335418	UDP-glycosyltransferase UGT5
**MSTRG.12404**	0.364723035	0.026226855	0.040029156	UDP-glycosyltransferase UGT4-like [*B. tryoni*]

Table S10. Expression pattern of esterases in response to ME in male *B. dorsalis* gut transcriptome

Symbol	log2(fc)	P Value	FDR	Description
**LOC105224146**	2.887913	0.002	0	juvenile hormone esterase-like
**LOC105224147**	3.362351	0.003	0	juvenile hormone esterase
**LOC105224154**	4.6005	0.004	0	juvenile hormone esterase-like
**LOC125778366**	3.529011	0.006	0	juvenile hormone esterase-like
**LOC105224709**	2.65391	1.2E-213	7.8E-212	sphingomyelin phosphodiesterase
**LOC105230320**	2.979631	9.9E-211	6.3E-209	esterase B1
**LOC105223265**	3.326921	3.5E-125	9.2E-124	esterase-5B
**LOC105227656**	3.686939	8.7E-120	2.2E-118	phosphotriesterase-related protein
**LOC105229622**	3.962168	1.2E-106	2.5E-105	juvenile hormone esterase
**LOC109579824**	2.645396	1.88E-98	3.78E-97	esterase B1
**LOC105222842**	2.258612	2.27E-78	3.5E-77	esterase B1
**LOC105226910**	6.797523	3.62E-66	4.67E-65	glycerophosphocholine phosphodiesterase GPCPD1
**LOC105223136**	-3.02314	9.38E-51	8.9E-50	ubiquitin thioesterase trabid
**LOC105231765**	16.17384	7.68E-32	4.73E-31	sphingomyelin phosphodiesterase 1
**LOC105224412**	1.239849	2.63E-29	1.5E-28	carboxylesterase 1E
**LOC125778446**	14.78025	3.87E-26	2.01E-25	juvenile hormone esterase
	-4.38748	1.03E-24	5.17E-24	Glycerophosphodiester phosphodiesterase 1 [*Eumeta japonica*]
**LOC105226911**	13.72618	3.31E-24	1.63E-23	glycerophosphocholine phosphodiesterase GPCPD1
**LOC105229967**	13.34124	1.76E-19	7.22E-19	juvenile hormone esterase
**LOC105224148**	0.673656	1.93E-19	7.91E-19	juvenile hormone esterase

**LOC109579590**	9.992827	7.92E-19	3.15E-18	putative glycerophosphocholine phosphodiesterase GPCPD1 homolog 2
**LOC105232714**	1.335206	1.13E-18	4.48E-18	sphingomyelin phosphodiesterase 4
**LOC105230316**	-3.42505	5.51E-18	2.12E-17	esterase B1
**LOC105231632**	-2.21756	1.15E-16	4.2E-16	acyl-coenzyme A thioesterase 9, mitochondrial
	8.781999	6.86E-16	2.43E-15	esterase B1 [*B. dorsalis*]
**LOC105231876**	1.697609	1.28E-15	4.47E-15	acetylcholinesterase
**LOC105228230**	-2.04472	5.91E-15	2E-14	2',5'-phosphodiesterase 12
**LOC105231548**	-2.74249	8.5E-14	2.75E-13	U6 snRNA phosphodiesterase 1
**LOC105231369**	-1.75695	1.42E-13	4.55E-13	protein phosphatase methylesterase 1
**LOC105229232**	-3.07819	8.43E-13	2.59E-12	probable cGMP 3',5'-cyclic phosphodiesterase subunit delta
**LOC105231766**	4.509472	2.12E-10	5.8E-10	sphingomyelin phosphodiesterase
**LOC105228087**	-1.6025	7.7E-10	2.04E-09	metallophosphoesterase 1
**LOC105223787**	-0.06444	7.88E-10	2.08E-09	glycerophosphocholine phosphodiesterase GPCPD1
**LOC105225932**	0.090277	1.86E-09	4.8E-09	lysosomal thioesterase PPT2 homolog
**LOC105224741**	-0.00067	2.77E-09	7.1E-09	acyl-protein thioesterase 2
**LOC105224905**	-0.12644	2.48E-08	6.04E-08	neuropathy target esterase sws
**LOC105232909**	-1.46021	1.12E-07	2.65E-07	ubiquitin thioesterase OTU1
**LOC105230338**	0.248148	2.44E-07	5.62E-07	esterase B1
**LOC105223794**	2.044847	4.73E-07	1.07E-06	glycerophosphocholine phosphodiesterase GPCPD1
**LOC105231880**	0.672491	1.23E-06	2.7E-06	esterase GA18864
**LOC105224085**	-4.79297	1.25E-05	2.58E-05	cAMP-specific 3',5'-cyclic phosphodiesterase
**LOC105233983**	-1.80992	8.47E-05	0.000165	metallophosphoesterase domain-containing protein 1
**LOC105230317**	-0.27889	0.000223	0.000418	esterase B1
**LOC105230236**	-1.59671	0.000479	0.000878	glycerophosphodiester phosphodiesterase 1
**LOC105227734**	0.09659	0.001156	0.002052	high affinity cAMP-specific and IBMX-insensitive 3',5'-cyclic phosphodiesterase 8
**LOC105224228**	0.072494	0.004415	0.007396	1-phosphatidylinositol 4,5-bisphosphate phosphodiesterase gamma-1
**LOC105230829**	0.031871	0.011227	0.017963	dual 3',5'-cyclic-AMP and -GMP phosphodiesterase 11
**LOC125775307**	7.345775	0.011696	0.018647	juvenile hormone esterase-like
**LOC105222723**	-1.03214	0.041822	0.061972	glycerophosphocholine phosphodiesterase GPCPD1
**LOC105232178**	0.398114	0.065738	0.094654	1-phosphatidylinositol 4,5-bisphosphate phosphodiesterase
**LOC105232119**	0.402902	0.083593	0.118294	high affinity cGMP-specific 3',5'-cyclic phosphodiesterase 9A,
**LOC105229367**	-1.28281	0.09724	0.135856	dual specificity calcium/calmodulin-dependent 3',5'-cyclic nucleotide phosphodiesterase 1
**LOC105233999**	-1.05464	0.107586	0.149105	acyl-coenzyme A thioesterase 13
**LOC105231364**	-2.24793	0.150624	0.203328	esterase B1-like
**LOC105230318**	-6.45396	0.295964	0.373672	esterase B1
**LOC105222388**	-1.44233	0.30531	0.384278	carboxylesterase 5A-like
**LOC105225795**	-1.14412	0.331524	0.413812	cGMP-specific 3',5'-cyclic phosphodiesterase
**LOC105224491**	-2.96829	0.445757	0.535115	palmitoleoyl-protein carboxylesterase NOTUM
**LOC125778681**	-8.24793	0.451274	0.539912	putative inactive carboxylesterase 4
**LOC105224440**	-1.63397	0.504423	0.594012	juvenile hormone esterase
**LOC105228217**	-1.92283	0.51357	0.602797	liver carboxylesterase 1
**LOC105231844**	-1.96253	0.566477	0.651415	esterase E4

**LOC105225886**	-0.9399	0.586041	0.668957	probable tyrosyl-DNA phosphodiesterase
**LOC105234124**	-0.58786	0.686414	0.762168	metallophosphoesterase 1 homolog
**LOC105232848**	-0.84148	0.879589	0.917826	1-phosphatidylinositol 4,5-bisphosphate phosphodiesterase classes I and II
**LOC105223755**	-0.79289	0.937153	0.9492	ubiquitin thioesterase otubain-like
**LOC105224915**	-0.80594	0.986802	0.988985	palmitoyl-protein thioesterase 1

Table S11. Expression pattern of HSPs in response to ME in male *B. dorsalis* gut transcriptome

Symbol	log2(fc)	P Value	FDR	Description
**LOC125778585**	1.728154	6.1E-124	2.2E-122	HSP 27
**LOC105231830**	2.263552	4.4E-98	1.11E-96	heat shock 70 kDa protein cognate 2
**LOC105223782**	1.183682	4.63E-64	6.9E-63	10 kDa HSP, mitochondrial
**LOC105229031**	0.911471	5.91E-60	8.17E-59	HSP 27
**LOC105229596**	0.498225	2.45E-48	2.67E-47	heat shock 70 kDa protein cognate 4
**LOC105224006**	0.957594	8.49E-48	9.16E-47	heat shock 70 kDa protein cognate 1
**LOC105222461**	-2.76888	9.57E-33	6.92E-32	HSP 23
**LOC105228080**	-4.07461	5.91E-31	4.05E-30	heat shock factor protein,
**LOC105226833**	-2.47846	8.27E-19	3.54E-18	activator of 90 kDa HSP ATPase homolog 1
**LOC105222460**	-4.53761	2.16E-14	7.58E-14	HSP 67B3
**LOC105222463**	2.333908	2.49E-14	8.72E-14	HSP 23
**LOC105222464**	0.596417	3.45E-14	1.2E-13	HSP 23
**LOC105233474**	9.41043	9.87E-11	2.88E-10	10 kDa HSP, mitochondrial-like
**LOC105228797**	-1.91059	2.08E-09	5.63E-09	HSP beta-1
**LOC105222465**	-2.64909	4.87E-09	1.29E-08	HSP 67B1
**LOC105225389**	-1.58151	4.04E-07	9.48E-07	heat shock 70 kDa protein cognate 5
**LOC105223445**	-1.75881	5.09E-07	1.19E-06	heat shock 70 kDa protein 4
**LOC125775805**	-0.06906	7.04E-07	1.62E-06	HSP 70-like
**LOC105224805**	-0.29228	5.2E-06	1.13E-05	heat shock 70 kDa protein 14
**LOC105231137**	-2.30932	2.62E-05	5.44E-05	heat shock factor-binding protein 1
**LOC125775804**	-0.16306	4.77E-05	9.69E-05	HSP 70
**LOC125775803**	-0.40634	0.000295	0.000561	HSP 70-like
**LOC125776475**	-2.12815	0.000458	0.000857	HSP 68-like
**LOC105222468**	-0.11526	0.000541	0.001007	HSP 27
**LOC125778668**	0.738437	0.00268	0.004666	HSP 23-like
**LOC105226434**	-0.85679	0.00433	0.007382	HSP 83
**LOC125778721**	1.340932	0.014116	0.022613	HSP23-like
**LOC125777106**	-0.53761	0.018431	0.029019	HSP75 kDa, mitochondrial
**LOC105222467**	0.746225	0.021233	0.033128	HSP23
**LOC125775806**	-0.64916	0.032448	0.049459	HSP 70-like
**LOC125775802**	-0.6821	0.043914	0.065618	HSP70-like
**LOC105233698**	-1.50989	0.061675	0.089951	HSP 68
**LOC125778667**	0.4639	0.067757	0.098156	HSP23-like
**LOC105225308**	-2.92081	0.070695	0.102079	HSP 27
**LOC105222466**	-0.67631	0.072912	0.10502	HSP23
**LOC105230159**	-0.9308	0.261336	0.334872	HSP75 kDa, mitochondrial
**LOC125775287**	-0.9225	0.560571	0.646031	HSP68-like

**LOC125775801**	-1.16829	0.70707	0.773431	HSP70-like
**LOC105230421**	-1.21612	0.725113	0.788861	HSP68
**LOC105222183**	-1.09866	0.897309	0.926575	60 kDa heat shock protein, mitochondrial

Table S12. Expression pattern of GR receptors in response to ME in male *B. dorsalis* legs transcriptome

Symbol	log2(fc)	P Value	FDR	Description
**LOC105226710**	7.584962501	0.139208457	0.215941242	GR and odorant receptor 21a-like
**LOC105232725**	0.673231952	0.15211809	0.233138222	GR and odorant receptor 63a
**LOC125776739**	-1.784122616	0.256609208	0.364325518	GR and odorant receptor 22-like
**LOC105232453**	-6.222392421	0.505569784	0.626641605	GR and odorant receptor 21a
**LOC125776742**	6.163230349	0.965285389	0.97531897	GR and odorant receptor 22-like
**LOC105225227**	2.74656551	5.78E-08	0.000000226	GR8a
**LOC105231250**	10.01448548	0.000117594	0.000315481	GR for bitter taste 22e-like
**LOC125779953**	-10.28154371	0.000355512	0.000889376	putative GR98b
**LOC109579632**	-9.577428828	0.005392039	0.011298365	putative GR98b
**LOC105224525**	-4.567897768	0.007794404	0.015852419	GR for sugar taste 43a
**LOC105229347**	-2.406400797	0.016877346	0.032302535	GR and pheromone receptor 32a
**LOC105223070**	7.702749879	0.046596551	0.081561206	putative GR28a
**LOC105222715**	8.279223644	0.085986938	0.14102931	putative GR58a
**MSTRG.3305**	1.65493087	0.135252049	0.210448095	GR2, partial [*Athetis dissimilis*]
**LOC105231253**	8.207827794	0.164829548	0.249334997	GR for bitter taste 22e-like
**LOC105223522**	7.510434522	0.167200712	0.252437501	GR and pheromone receptor 33a
**LOC115066260**	-2.657531127	0.21180182	0.309343566	putative GR98b
**LOC105223121**	-0.056659512	0.250320343	0.356788823	putative GR28b
**LOC105225721**	6.930737338	0.290171658	0.404122108	GR for bitter taste 66a-like
**LOC105233094**	-6.464886049	0.301958881	0.416867747	GR for sugar taste 64a
**LOC115066475**	-6.934673752	0.301958881	0.416867747	GR and pheromone receptor 32a-like
**LOC105225214**	-1.343265271	0.333180782	0.452012395	GR8a
**MSTRG212970**	-9.288481612	0.346361014	0.4659736	predicted: GR and pheromone receptor 32a-like [*Musca domestica*]
**LOC125778996**	-6.845490051	0.505569784	0.626641605	putative GR77a
**LOC105230597**	-1.45222597	0.512579476	0.633338666	putative GR2a
**LOC105231252**	6.882643049	0.521311415	0.642617181	GR for bitter taste 22e-like
**MSTRG21297**	-2.352439399	0.529029916	0.650155227	putative GR28b [*B. tryoni*]
**LOC115066509**	6.906890596	0.562239656	0.675068121	putative GR22d
**LOC105233089**	6.254241287	0.563678513	0.676109318	GR for sugar taste 64e
**LOC105233098**	6.403722186	0.579113015	0.690943414	GR5a for trehalose
**LOC115066667**	6.094517599	0.670576783	0.772344239	GR for sugar taste 64f
**LOC105228990**	6.169925001	0.676064569	0.777466037	putative GR47b
**LOC105229094**	-1.261423356	0.69183924	0.790991903	GR for bitter taste 66a
**LOC105229190**	0.338983259	0.692500163	0.791428757	putative GR59f
**LOC105227650**	-1.604862058	0.701708207	0.797023189	putative GR98b
**MSTRG3804**	-0.010694601	0.762344554	0.848431141	putative GR28b [*B. tryoni*]
**LOC105230819**	5.48112669	0.771847668	0.855710228	GR68a
**LOC105230818**	5.273018494	0.853203346	0.910344048	GR and pheromone receptor 32a-like,
**LOC105224551**	-0.987790942	0.869043792	0.910344048	GR for sugar taste 43a
**LOC105225752**	3.624490865	0.871728834	0.910344048	putative GR93c
**LOC105228213**	4.321928095	0.871728834	0.910344048	putative GR22a
**LOC105228623**	3.969626351	0.871728834	0.910344048	GR23a

**LOC105233095**	4.087462841	0.871728834	0.910344048	GR for sugar taste 64b
**LOC105223308**	4.857980995	0.961922622	0.974557814	putative GR39b
**LOC105225869**	5.273018494	0.961922622	0.974557814	putative GR89a
**LOC115066510**	5.222392421	0.968675889	0.975411396	putative GR22b
**LOC125778345**	5.273018494	0.968675889	0.975411396	putative GR39b
**LOC125779736**	5.26052755	0.968675889	0.975411396	putative GR22a
**LOC109579940**	-0.540403831	0.971489086	0.977619875	putative GR94a

Table S13. *Bdor94a*-like orthologous in various insect species and their functions

Species	NCBI symbol	Reference sequence NO; mRNA and Protein(s)	Function & references
Bactrocera dorsalis (94a-like)	LOC105225117	XM_011203454.1	ME receptor
Bactrocera tryoni (94a-like)	LOC120770124	XM_040097304.1	Odorant receptor
Drosophila melanogaster (94a)	FLYBASE:FBgn0039033	NP_524455.1 http://flybase.org/reports/FBgn0039033.html	(Carey & Carlson, 2011) chemoreceptor that mediates response to volatile chemicals 28821769,25619769, 23690583, 16332533, 16139208, 15882644, 12782114, 10943836
Adelges cooleyi (94a-like)	LOC126845874	XP_050440819.1	enables olfactory receptor activity and odorant binding
Aedes albopictus (94a-like) (Foshan strain) Adult male	PRJNA1069059	XM_062857073.1	
Bradysia coprophila (94a-like)	LOC119067002; LOC119085435	NCBI Gene (ID 119067002 XM_037169733.1 XP_037025628.1	Sensory perception of odorant
Spodoptera litura (94a-like)	LOC111356232	XM_022970521.1	
Lucilia sericata (94a-like)	LOC119612783	XM_037968652.1	
Wyeomyia smithii (94a-like)	LOC129729229; LOC129729228; PRJNA961728	XM_055687727.1	
Diabrotica virgifera virgifera (94a0-like)	LOC126888542	XM_050656888.1	
Hermetia illucens (94a-like)	LOC119655231; LOC119656814	XM_038061013.1	
Cydia strobilella (94a-like)	LOC134755433	XM_063691990.1	
